# Cation-selective two-dimensional polyimine membranes for high-performance osmotic energy conversion

**DOI:** 10.1038/s41467-022-31523-w

**Published:** 2022-07-08

**Authors:** Zhen Zhang, Preeti Bhauriyal, Hafeesudeen Sahabudeen, Zhiyong Wang, Xiaohui Liu, Mike Hambsch, Stefan C. B. Mannsfeld, Renhao Dong, Thomas Heine, Xinliang Feng

**Affiliations:** 1 Center for Advancing Electronics Dresden (cfaed) and Faculty of Chemistry and Food Chemistry, Technische Universität Dresden, 01062 Dresden, Germany; 2 Max Planck Institute of Microstructure Physics, Halle (Saale), 06120 Germany; 3 Suzhou Institute for Advanced Research, University of Science and Technology of China, 215123 Suzhou, Jiangsu China; 4 School of Chemistry and Materials Science, University of Science and Technology of China, 230026 Hefei, Anhui China; 5 Center for Advancing Electronics Dresden (cfaed) and Faculty of Electrical and Computer Engineering, Technische Universität Dresden, 01062 Dresden, Germany; 6 Helmholtz Center Dresden-Rossendorf, Institute of Resource Ecology, Leipzig Research Branch, Permoserstr. 15, 0416 Leipzig, Germany; 7 Department of Chemistry, Yonsei University, Seodaemun-gu, Seoul 120-749 Republic of Korea

**Keywords:** Porous materials, Design, synthesis and processing, Synthesis and processing

## Abstract

Two-dimensional (2D) membranes are emerging candidates for osmotic energy conversion. However, the trade-off between ion selectivity and conductivity remains the key bottleneck. Here we demonstrate a fully crystalline imine-based 2D polymer (2DPI) membrane capable of combining excellent ionic conductivity and high selectivity for osmotic energy conversion. The 2DPI can preferentially transport cations with Na^+^ selectivity coefficient of 0.98 (Na^+^/Cl^−^ selectivity ratio ~84) and K^+^ selectivity coefficient of 0.93 (K^+^/Cl^−^ ratio ~29). Moreover, the nanometer-scale thickness (~70 nm) generates a substantially high ionic flux, contributing to a record power density of up to ~53 W m^−2^, which is superior to most of nanoporous 2D membranes (0.8~35 W m^−2^). Density functional theory unveils that the oxygen and imine nitrogen can both function as the active sites depending on the ionization state of hydroxyl groups, and the enhanced interaction of Na^+^ versus K^+^ with 2DPI plays a significant role in directing the ion selectivity.

## Abstract (web summary)

Two-dimensional (2D) membranes are emerging candidates for osmotic energy conversion but the trade-off between ion selectivity and conductivity remains the key bottleneck. Here, the authors demonstrate a fully crystalline imine-based 2D polymer membrane capable of combining excellent ionic conductivity and high selectivity for osmotic energy conversion.

## Introduction

Membrane-based reverse electrodialysis is a future technology to capture the “blue” osmotic energy between seawater and fresh water for large-scale industrial and domestic electricity supply. It can also be synergistically coupled with other technologies such as desalination, water splitting, and microbial fuel cells^1^. Additionally, small-scale reverse electrodialysis setup can be used to power implantable device by utilizing different streams inside the body, or integrated as portable power source for paper-based analytical devices^2,3^. As the core component in reverse electrodialysis, traditional membranes such as commercial ion-exchange membranes, suffer from inadequate ion transport abilities, leading to commercially unviable power output of around 2 W m^−2,^^4^. Two-dimensional (2D) materials have emerged as promising building blocks to develop alternative functional membranes^5–7^. Examples include nanoporous laminar membranes formed by stacking atomically thin nanosheets such as transition metal carbide (MXene) and boron nitride, and single-layer 2D materials based membranes such as single-pore MoS_2_ and porous graphene^8–16^. The former has the advantages of high selectivity, easy fabrication and excellent up-scalability, but shows limited ionic conductivity caused by the large thickness from several to tens of micrometers and disordered ion transport pathways. The latter can address this issue to allow for ultrahigh conductivity, however, inevitably sacrificing its own ion selectivity due to the poor controllability over the pore dimension and distribution, the low density of functional groups, and the quantities of stochastic defects. Therefore, it remains a formidable challenge to overcome the trade-off effect between the ion selectivity and conductivity. 2D polymers (2DPs) are covalently linked networks of monomers with periodic bonding in two distinct dimensions^17^. On-water surface synthesis has been recently demonstrated to provide large-area highly crystalline 2DPs with thickness from single-layer to multi-layers^18,19^. Such 2DPs with excellent stability can be isolated as robust ultrathin nanoporous membranes with high densities of intrinsic mono-disperse pores, high density of functional groups, and good structure uniformity, appearing to be great candidates to overcome the aforementioned trade-off effect^20,21^.

Here, we demonstrate a fully crystalline, nanometer-thick imine-based 2DP (2DPI) membrane for osmotic energy conversion with outstanding performance. Thanks to its intrinsic mono-disperse pores (~10^17^ m^−2^), high spatial density of functional hydroxyl groups (~1.5 × 10^27^ m^−3^), and fully crystalline structure, the 2DPI membrane is capable of coupling excellent ionic conductivity and high selectivity together, contributing to a record power density of 53 W m^−2^ that is one order of magnitude higher than traditional ion-exchange membranes and superior to most of nanoporous 2D membranes^12,14,15,22–26^. We achieve the molecular-level understanding of the energy conversion process by calculating the interactions of solvated ions with the 2DPI backbone and their diffusion barriers using density-functional theory (DFT). The achievement will spark further efforts to rationally design crystalline 2DP membranes for nanofluidic energy applications.

## Results

### Fully crystalline 2DPI membrane

**Fig. 1:**
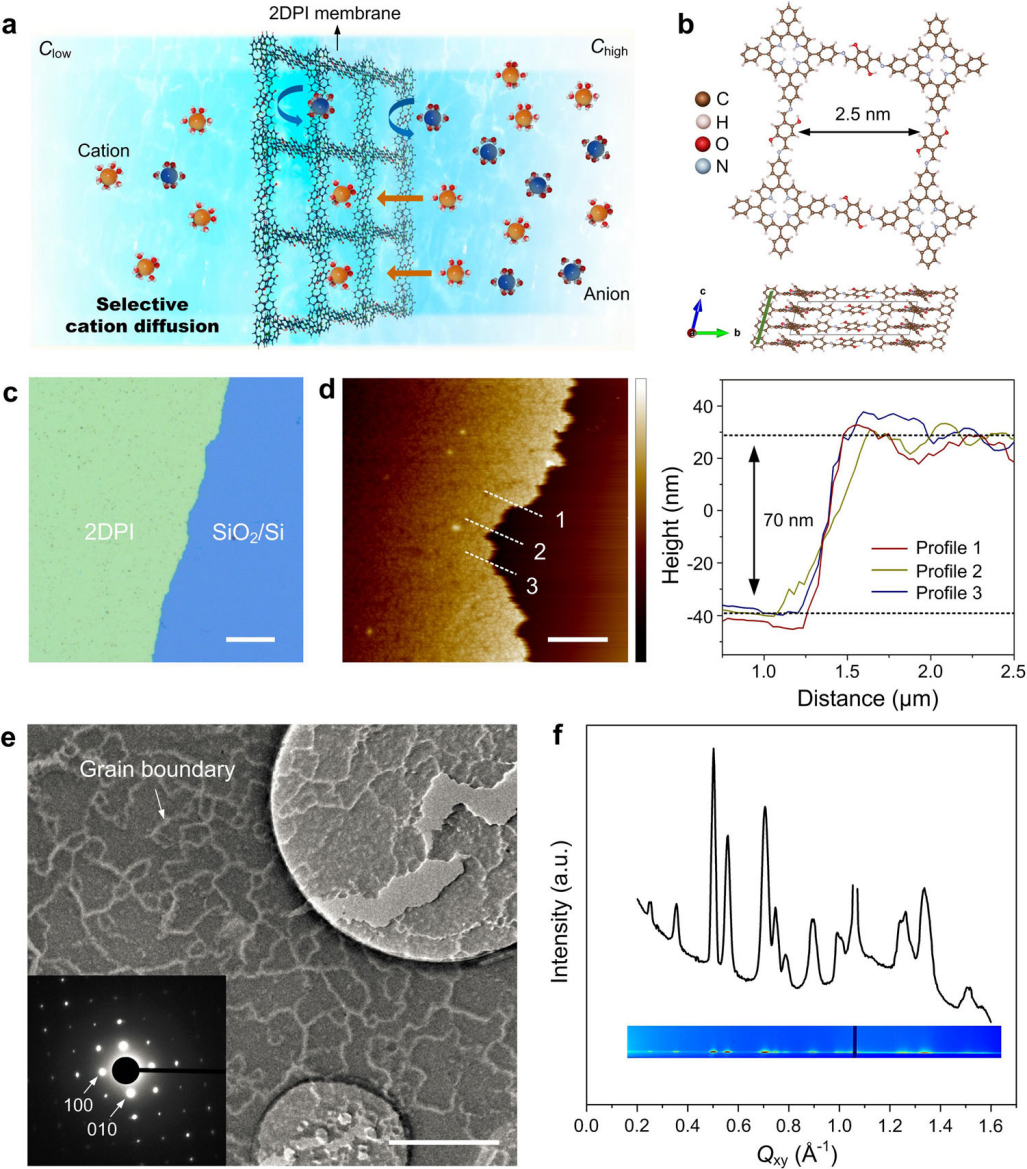
Ultrathin fully crystalline 2DPI membrane. **a** Schematic of the selective ion diffusion process across the 2DPI membrane. **b** The structure of the 2DPI membrane as well as its most stable stacking mode (inclined AA stacking). **c** The optical microscopy image of the 2DPI membrane (scale bar: 50 µm). **d** AFM of the 2DPI membrane on 300 nm SiO_2_/Si, revealing an ultrathin thickness of about 70 nm (scale bar: 300 nm; color bar: −59.2 to 66.8 nm). **e** TEM images and SAED pattern of the 2DPI membrane, indicating its excellent crystallinity on the microscale (scale bar: 800 nm). **f** In-plane intensity profile of a 2DPI membrane. The inset shows the in-plane peaks from the 2D GIWAXS pattern.

The ultrathin 2DPI membrane was prepared by the surfactant-monolayer-assisted interfacial synthesis (SMAIS) method (see “Methods”) (Fig. 1a, b and Supplementary Fig. 1)^19^. The as-prepared membrane is homogeneous with uniform color and a clearly visible edge (Fig. 1c and Supplementary Fig. 2). The average thickness is ~70 nm as characterized by atomic force microscopy (Fig. 1d). The imine bond formation is confirmed by the Raman spectra (Supplementary Fig. 3) and the extended conjugation is evidenced by the red shift of the S band in the UV–vis spectra (Supplementary Fig. 4). X-ray photoelectron spectroscopy confirms the existence of functional hydroxyl groups as well as the imine nitrogen (Supplementary Fig. 5). Transmission electron microscopy reveals that the membrane is fully crystalline without amorphous regions and the domain sizes range from 0.1 to 1 μm. A single-crystalline square lattice with a spacing of 25 Å is observed in the selected-area electron diffraction (SAED) (Fig. 1e). Furthermore, the crystallinity on the macroscopic level was investigated by grazing-incidence wide-angle X-ray scattering (GIWAXS) measurement (Fig. 1f and Supplementary Fig. 6). Sharp and discrete Bragg peaks are visible in the GIWAXS scattering pattern near *Q*_Z_ = 0 at the Yoneda peak position but with only faint tails in the vertical direction. This indicates that there is a high degree of 2D in-plane crystallinity but a much lower degree of coherence in the vertical direction, a situation comparable to turbostratic graphene. The in plane peaks of 0.25 Å^−1^ and 0.5 Å^−1^ correspond to the Bragg reflections of a square lattice (100) and (200) with *a* = *b* = 25.3 Å. The broad out-of-plane signal at *Qz* = 1.49 Å^−1^ can be assigned to the interlayer π–π stacking of ~4.2 Å, agreeing well with the first-principles calculations (Supplementary Figs. 7, 8).

### Surface-charge-governed ion transport

**Fig. 2:**
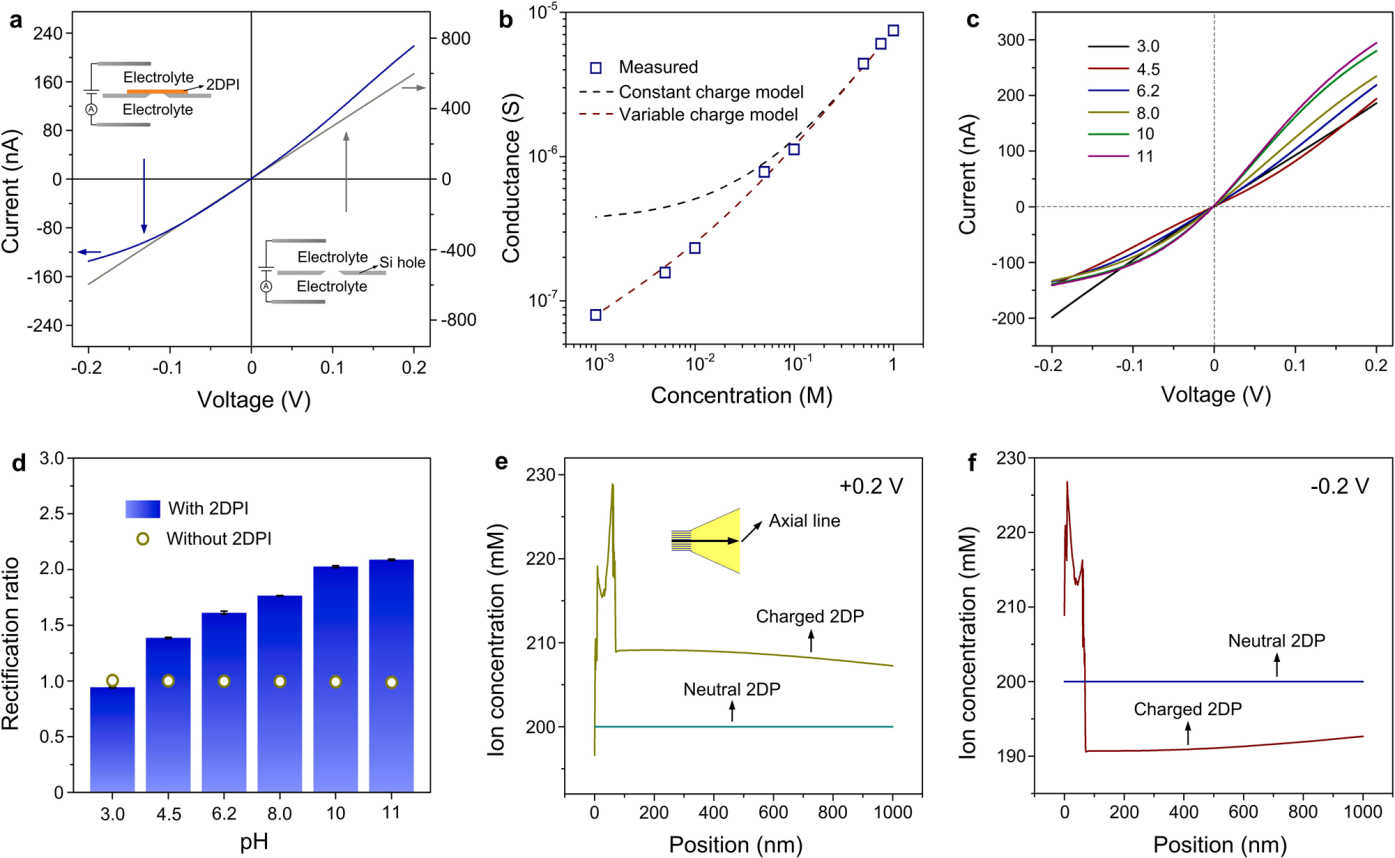
Surface-charge-governed ion transport. **a** *I–V* curves of the silicon hole before and after deposition with 2DPI membrane recorded in 0.1 M KCl. **b** Transmembrane conductance as the function of electrolyte concentration. The red line denotes the fitted values with variable surface charge density and the black line represents values assuming constant surface charge –12 mC m^−2^. **c** *I–V* curves of the 2DPI membrane recorded in 0.1 M KCl of different pH values. **d** Variations of the rectification ratio of the silicon hole before and after deposition with 2DPI membrane in response to pH value. **e**, **f** The total ion concentration on the axial line of two representative models that simulate the neutral 2DP deposited substrate and charged 2DP deposited substrate under +0.2 V (**e**) and −0.2 V (**f**). Error bars represent s.d.

The transmembrane ionic transport properties of the 2DPI membrane were examined by current−voltage (*I*−*V*) measurements (Supplementary Fig. 9)^27^. Potassium chloride (KCl) was selected as the standard electrolyte because of the close diffusion coefficients of K^+^ and Cl^−^ ions^28^. The 2DPI membrane was supported on a silicon wafer which contains an open hole of about 12 µm^2^ (Supplementary Fig. 10). As shown in Fig. 2a, the blank silicon hole exhibits a linear *I–V* curve. After deposition with 2DPI, obvious ionic rectification evidenced by a non-linear *I–V* curve is observed. Such a non-linear transport behavior can be ascribed to the surface charge effect of 2DPI membrane^29^. The corresponding rectification ratio, quantified by the ratio of current measured at positive potential (+0.2 V) and negative potential (−0.2 V), can reach approximately 1.62. The transmembrane ionic transport exhibits a surface-charge-governed behavior (Fig. 2b): the ionic conductance follows a linear rule at high concentration and gradually deviates upon decreasing electrolyte concentration (<0.05 M). The surface charge density can be evaluated by fitting the data with the existing conductance model^30^. From the measured conductance for KCl 0.1 M, a value of about –12 mC m^−2^ was obtained (see “Methods” and Supplementary Fig. 11), which is higher than conventional 2D materials such as graphene oxide, clay, and boron nitride which are in the range of 0.5‒2 mC  m^−2,^^31,32^. This apparent surface charge density is much lower than the intrinsic charge density of the fully deprotonated 2DPI of about –90 mC m^−2^, which can be ascribed to insufficient deprotonation at pH 6.2^33^.

The ionic transport shows a strong dependence on the pH value of the electrolyte. At pH 3, the *I–V* curve is nearly linear with very weak rectification of about ~0.94, but in the reverse direction. As the pH increases from 3 to 11, the ionic current at −0.2 V stays nearly unchanged about 130 nA, while the ionic current at +0.2 V gradually increases from 190 to 290 nA (Fig. 2c). Overall, the corresponding rectification ratio increases from 0.9 to 2.1, which is distinct from the separate silicon micro-channel before 2DPI coating whose rectification ratio is about 1.0 and shows the minimal response to pH changes (Fig. 2d and Supplementary Fig. 12). The variation of rectification behavior indicates the increase of negative surface charge upon increasing pH which can be ascribed to the gradual deprotonation of hydroxyl groups in 2DPI. Note that the slight inverted rectification at pH = 3 is owing to the partial protonation of nitrogen^34^ in imine and porphyrin units that generates an excess positive charge. The change of surface charge is also confirmed by the Zeta potential measurement of 2DPI membrane (Supplementary Fig. 13), which shows a similar trend with the rectification results. The experimentally observed ionic rectification was also investigated using continuum–based Poisson–Nernst–Planck (PNP) model, which has been validated to be an effective tool to simulate the ion transport through nanochannel membranes^35^. Two representative models that simulate the neutral 2DP deposited substrate and charged 2DP deposited substrate were established (“Methods”; Supplementary Figs. 14–15), which contains about 1100 2.32 nm-wide 2DP channels (inter-channel distance: 2.56 nm) connected by one 3000 nm-wide silicon conical channel. It is shown that positive ionic rectification can be observed, evidenced by ion enrichment (Fig. 2e) at the positive bias and ion depletion (Fig. 2f) at the negative bias in the interface, only if the 2DP membrane is negatively charged, further confirming our hypothesis that the surface charge of 2DPI plays the dominant role in the ion transport. The experimentally observed ionic rectification is also in line with that observed in unipolar diode which contains a zone that is neutral and a zone that is charged^36,37^.

### Selective ion diffusion and osmotic energy conversion

**Fig. 3:**
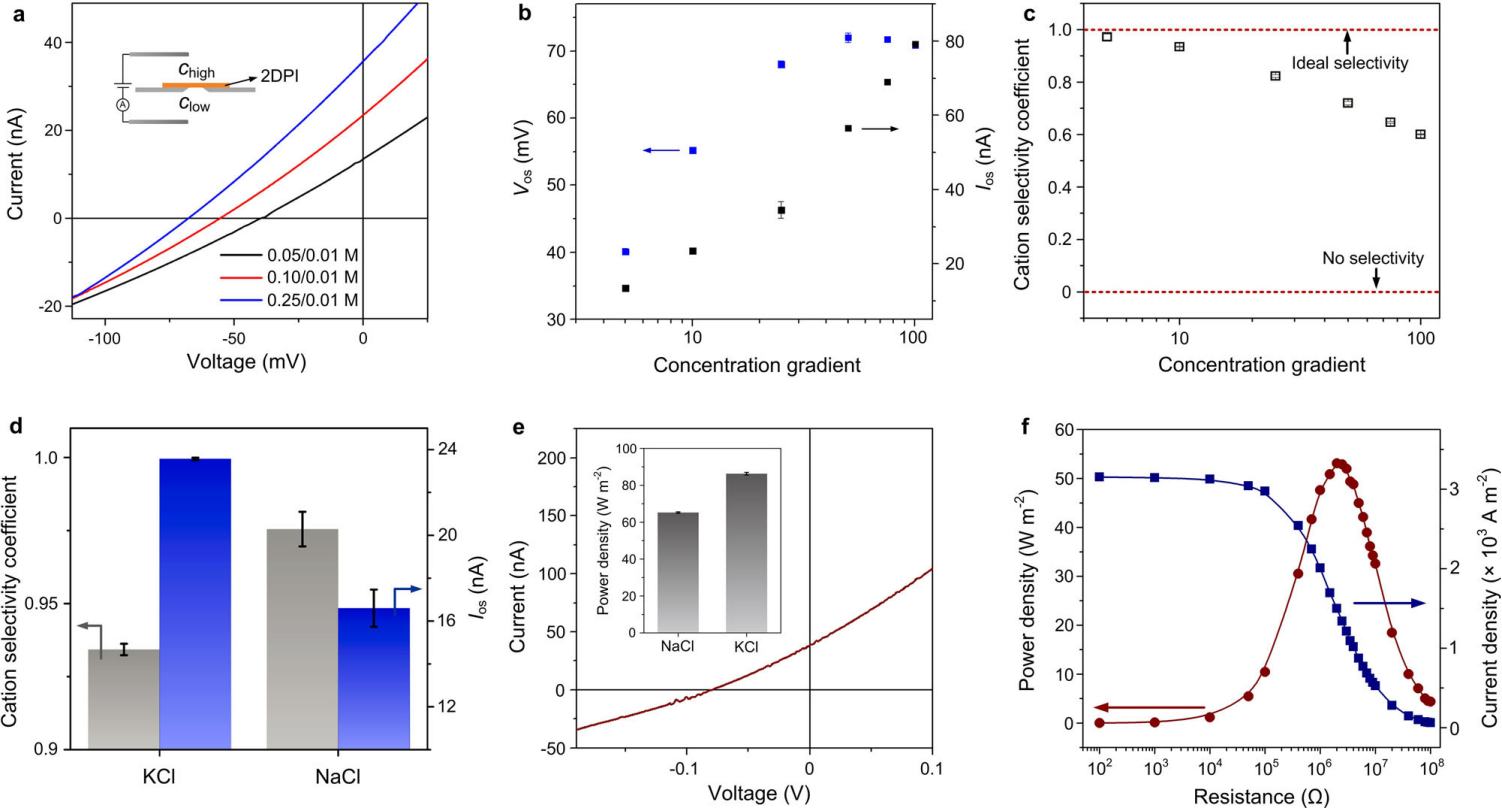
Selective ion diffusion and osmotic energy conversion. **a** *I–V* curves of the 2DPI membrane recorded under a series of KCl concentration gradient (the low concentration side, *c*_low_, is set to 0.01 M). **b** Variations of *V*_os_ and *I*_os_ as a function of KCl concentration gradient. **c** The ion selectivity coefficient as a function of KCl concentration gradient. **d** Comparison of the ion selectivity coefficient and *I*_os_ in NaCl and KCl electrolyte with a concentration gradient of 10-fold. **e** *I–V* curve of the 2DPI membrane recorded under 0.5/0.01 M NaCl. (Inset: comparison of the power density in KCl and NaCl of same concentration gradient, 0.5/0.01 M). **f** Power generation on external circuit by mixing artificial river water and seawater. Error bars represent s.d.

Afterwards, we investigated the transmembrane ion diffusion across the 2DPI membrane by applying an electrolyte concentration gradient. The negatively charged 2DP network will selectively transport K^+^ ion, creating a net potential difference across the membrane^38,39^. The intercepts on the voltage and current axis allow us to find the corresponding osmotic potential (*V*_os_) and osmotic current (*I*_os_), respectively (Fig. 3a). In order to eliminate the influence of redox potential produced by the unequal voltage drops on the electrodes, standard saturated Ag/AgCl salt bridge electrodes are used^40^. The blank silicon hole is not ion selective and the *I*_os_ and *V*_os_ are both close to zero regardless of the concentration gradient (Supplementary Fig. 16). For the 2DPI membrane, the measured *I*_os_ increases from 13 to 80 nA as the concentration gradient increases from 5 to 100, while *V*_os_ first increases from 40 to 72 mV, and then decreases at high concentration gradient ratio >50 (Fig. 3b). The corresponding ion selectivity coefficient^41,42^, *S* (calculated by the measured *V*_os_, where *S* = 0 refers to the non-selective case and *S* = 1 refers to the ideal cation-selective case, see “Methods”), decreases from 0.98 to 0.6 upon increasing the concentration gradient from 5 to 100 (Fig. 3c). The decrease of *S* can be ascribed to the decrease of Debye length in the high concentration side which can weaken the ion selectivity of the membrane^43^. Note that if we reduce the testing size to sub-micrometer-scale (~0.33 µm^2^), the decrease of ion selectivity under high concentration gradient will disappear (Supplementary Fig. 17). This is because, upon decreasing the membrane size, the effect of grain boundary and defect will be largely weakened, and the *S* in all concentration gradient are above 0.8.

The selective ion diffusion process is observed to be pH dependent due to the variation of surface charge density. At pH = 3, the 2DPI membrane exhibits a slight Cl^−^ selectivity, manifested by a positive *V*_os_ of about +2 mV (Supplementary Fig. 18), in agreement with the results in Fig. 2d. The selective ion diffusion was also investigated using NaCl electrolyte. Compared with the *S* of K^+^ ion of about 0.93 (K^+^/Cl^−^ selectivity ratio ~29), the Na^+^ ion exhibits a substantially higher *S* of about 0.98, which corresponds to a Na^+^/Cl^−^ selectivity ratio of about 84. Under similar testing conditions, the observed ion selectivity coefficient outperforms those previously reported for ultrathin organic and inorganic materials such as single-layer MoS_2_ and holey-graphene-like membranes (Supplementary Table 1)^9,44^. We also note that although K^+^ ion exhibits a lower *S* compared to Na^+^ ion, the corresponding *I*_os_ in KCl is much larger (Fig. 3d).

The osmotic energy conversion behavior was evaluated by comparing the theoretical output power density, *P*_max_ = *I*_os_ × *V*_os_/(4 × *A*), where *A* is the working area. Under a 10-fold concentration gradient of pH 6.2, the *P*_max_ in KCl is 27.6 W m^−2^, which is higher than that in NaCl of 20.3 W m^−2^, which can be ascribed to the larger *I*_os_ in KCl electrolyte. We also note a substantial decrease of power output in multivalent electrolyte (i.e. CaCl_2_ of 0.46 W m^−2^ and AlCl_3_ of 1.22 W m^−2^) (Supplementary Fig. 19). This is because the transport of multivalent ions will be largely hindered due to their larger hydrated size^40^. Especially, in AlCl_3_ electrolyte, the 2DPI membrane even changes into anion-selective (evidenced by a positive *V*_os_ of 26 mV) and Cl^−^ becomes the dominating ionic current carrier. Next, we evaluate the osmotic energy conversion behavior with seawater and river water (0.5/0.01 M NaCl) (Fig. 3e). The power density can achieve 65.2 W m^−2^ with an internal resistance of 2 MΩ, which is superior to most of the nanoporous 2D membranes such as laminar MXene, boron nitride composite, and single-layer porous graphene (0.8–35 W m^−2^) (Supplementary Table 2)^12,14,15,22–26^. The generated power can be output to external circuit to supply an electronic load. As the resistance increases, the current density on the external circuit decreases accordingly. The output power density, calculated as *P*_out_ = *I*^2^ × *R*, achieves a maximum value of 53.1 W m^−2^ (Fig. 3f). Note that the *P*_max_ in KCl electrolyte (0.5/0.01 M) is still the highest due to its much larger *I*_os_, reaching a value of about 86.2 W m^−2^ (Supplementary Fig. 20).

Furthermore, the influence of testing membrane area on the power output is investigated. In detail, *S* of sub-micrometer-scale device can achieve ~1, indicating ideal cation selectivity, while *S* maintains stable around 0.68 upon increasing the working area (Supplementary Fig. 21). The power density of the sub-micrometer-scale device can reach approximately 130 W m^−2^, and further increasing the membrane area will cause a decrease of power density to 44 W m^−2^ (Supplementary Fig. 22). The observed power attenuation upon scaling up can be ascribed to the combined effect of multiple factors such as entering resistance, hindered counter-ion diffusion, and increased stochastic defects and grain boundaries. In the future, efforts such as suppressing entering resistance, increasing ion selectivity, and optimizing synthetic protocols to obtain larger crystalline size are promising routes. The 2DPI membrane also exhibits good stability. For the lifetime testing, *V*_os_ and *I*_os_ are monitored every 12 h for 15 days. The measured *V*_os_ maintains stable, indicating that the membrane could effectively stabilize transmembrane concentration gradient and promote continuous osmotic energy harvesting. The measured *I*_os_ undergoes a 15.8% decrease, implying the increasing ionic resistance, which can be ascribed to many factors such as charge polarization and structure deterioration of the membrane. As a result, the power density only undergoes 12.7% decrease (Supplementary Fig. 23), demonstrating the good stability of the membrane, which can be ascribed to the high stability of imine bonding under neutral pH condition.

### Ion diffusion mechanism

**Fig. 4:**
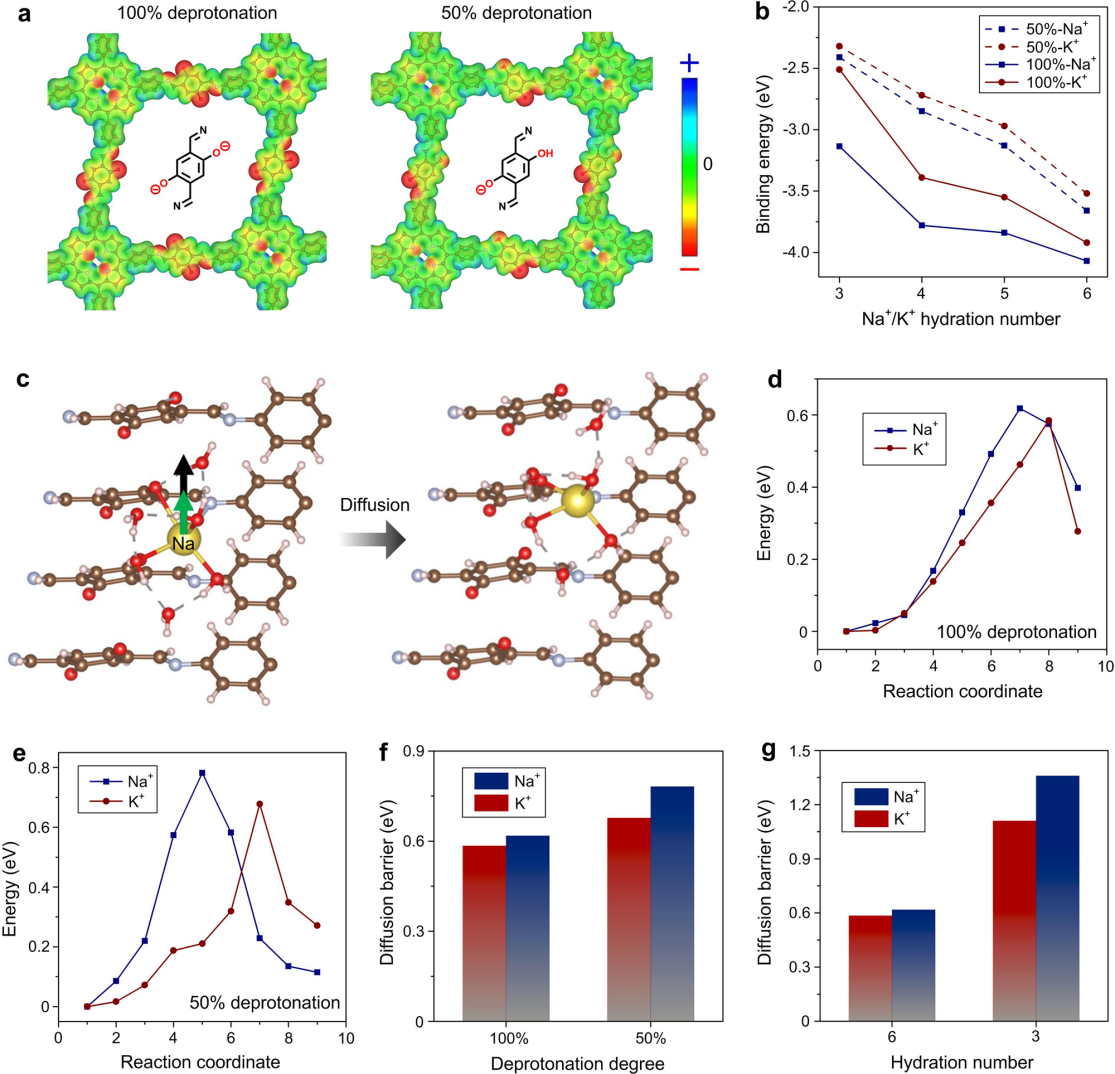
Ion diffusion mechanism revealed by DFT calculations. **a** Electrostatic potential (ESP) surface (isosurface value: 0.01 e.Å^−3^) of fully (100%) deprotonated and partially (50%) deprotonated 2DPI. The blue and red colors denote less and more electron density in the ESP surface, respectively. For the fully deprotonated 2DPI, electrons are strongly localized on oxygen anions followed by imine nitrogen, while the electrons are more concentrated on the deprotonated oxygen in the partially deprotonated 2DPI. **b** Binding energy of K^+^ and Na^+^ ions with different hydration numbers on fully and partially deprotonated 2DPI surfaces. **c** Pathways for Na^+^ ion diffusion through fully deprotonated 2DPI. The analyzed pathway (denoted by green color) involves the diffusion from the most stable site (2O-1N: hydrated ions occupy the interlayer space) to an intermediate metastable site (1O-1N: hydrated ions bind at the edge of the layer), through which the hydrated ion passes to reach its stable neighboring identical site (black color). **d**, **e** Energy profile of diffusion path for K^+^ and Na^+^ ions through fully **d** and partially **e** deprotonated 2DPI. **f** Comparison of the diffusion barriers of K^+^ and Na^+^ ions through fully and partially deprotonated 2DPI. **g** Diffusion barriers of K^+^ and Na^+^ ions with different hydration number through fully deprotonated 2DPI.

Atomistic understanding of the ion diffusion process is obtained by a series of DFT calculations (see “Methods”; Supplementary Figs. 7-8, 24–33). The linkers in 2DPI can be charged by hydroxyl group ionization, with one or with both hydroxyl groups being deprotonated (later-on referred to as partially and fully deprotonated, respectively, see Fig. 4a). For example, we discuss a system where the cations have been hexahydrated at the beginning, which yields the same overall results as the model systems with tri-, tetra-, and penta-hydrated cations (Supplementary Figs. 26, 27). For the fully deprotonated 2DPI, electrostatic potential (ESP) plot indicates that electrons are strongly localized on oxygen anions (Fig. 4a) followed by imine nitrogen. The K^+^ ion is observed to be preferentially coordinated by two oxygens and one more imine nitrogen, represented by the binding site 2O-1N (Supplementary Fig. 24). In the most stable binding mode, the inner and outer hydration shells are formed by four and two water molecules, respectively (denoted as 2O-1N:4W + 2W, Supplementary Fig. 24(h)). For the partially deprotonated 2DPI, the electrons are more concentrated on the deprotonated oxygen. In this case, the inner hydration shell remains the same; however, the preferential binding site changes to 2O (Supplementary Fig. 25) as one imine nitrogen is already involved in the H-bonding with the hydroxyl group and the other imine nitrogen exceeds the bonding region of hydrated ions due to the structural arrangement of 2DPI layers. Overall, in both cases, the direct binding of Na^+^/K^+^ is favored regardless of their hydration number (Supplementary Figs. 26, 27), with stronger binding energy for Na^+^ compared to K^+^, which plays a significant role in directing the ion selectivity (Fig. 4b).

The ion diffusion through the 2DPI membrane is analyzed by calculating the diffusion barriers in the out-of-plane direction (see Fig. 4c and Supplementary Fig. 28). For the fully deprotonated polymer, the diffusion barrier of Na^+^ ion (0.62 eV) is higher than that of the K^+^ ion (0.58 eV) (Fig. 4d). Possible reasons for this are: (1) the interaction of Na^+^ ion with the 2O-1N binding site is stronger and thus requires a higher activation energy for Na^+^ ion to diffuse through the path, and (2) the bond length of K-O/N (2.74/2.98 Å) (O/N of 2DPI) is larger than that of Na (2.70/2.59 Å), providing increased flexibility in the coordination of K^+^ ion with active site, which lowers the activation barrier for its diffusion. Overall, this sluggish diffusion decreases the Na^+^ ion mobility compared with K^+^ ion, resulting in the lower current observed in NaCl. For the partially deprotonated polymer, the energy barriers follow the same trend (Na^+^ ion of 0.78 eV and K^+^ ion of 0.68 eV), but with higher values compared to fully deprotonated system (Fig. 4e, f). We attribute these higher energies to the fact that in partially deprotonated 2DPI, the Na/K ion passes through a transition state which involves direct binding of ion with only one of the available active sites (1O site in Supplementary Fig. 29). This destabilizes the intermediate states in the partially deprotonated 2DPI, while in fully deprotonated case, cations maintain a direct binding state with two of the negatively charged active sites (either 2O or 1O-1N) throughout the constructed diffusion paths (Supplementary Fig. 28).

A comparative diffusion analysis based on the fully deprotonated models with hydration number 3 (this refers to the typical case where the ion has only the first inner hydration shell (Supplementary Fig. 30-31)) reveals that the diffusion barrier is also higher for Na^+^ (1.36 eV) than for K^+^ (1.11 eV). However, we note that there is an overall increase of energy barrier difference to 0.25 eV compared to the hexahydrated 2DPI (Fig. 4g). This overall increase in the diffusion barrier for hydration number 3 suggests that the second hydration shell plays an important role in stabilizing the intermediate structures and lowering the net diffusion barrier. Besides the monovalent ions, we also simulated divalent Ca^2+^ ion. A much higher diffusion barrier of about 0.99 eV is obtained (Supplementary Fig. 32) compared to Na^+^ (0.62 eV) and K^+^ (0.58 eV) ions, indicating that its transport will be largely hindered, in agreement with the ultralow power output measured for CaCl_2_. In the diffusion path, the Ca^2+^ will undergo several different binding states. In the high energy intermediate state, its inner hydration shell loses one H_2_O molecule, and the coordination number reduces from 7 to 5 as the binding site also changes from 2O-1N to 1O-1N (Supplementary Fig. 33). This unfavorable five coordinated intermediate structure results in the increased energy barrier.

In summary, we have experimentally and theoretically investigated the ion transport and osmotic energy conversion of a fully crystalline, nanometer-thick 2DPI membrane. Benefiting from its intrinsic mono-disperse pores (~10^17^ m^−2^), high spatial density of hydroxyl groups (~1.5 × 10^27^ m^−3^), fully crystalline structure, and ultrathin thickness, the 2DPI membrane is capable of coupling excellent ionic conductivity and high selectivity together. As a result, a substantially high power density of about 53 W m^−2^ is achieved in the condition of mixing river water and seawater that is one order of magnitude higher than traditional ion-exchange membranes and superior to most of the nanoporous 2D membranes. This work as a basic platform could potentially spark further experimental and theoretical efforts in the design of thin-film crystalline 2DPs for osmotic energy conversion. We would also like to mention that the 2DP membranes using SMAIS method are upscalable. Nevertheless, there is still a long way to go for large-scale applications because an enormous membrane area will be needed for real reverse electrodialysis plot-scale setup. The performances for real-size application need to be evaluated. On the other hand, the molecular understanding of the ion transport process in 2DP membranes will also provide significant guidance for other related fields such as ion sieving, supercapacitors, and lithium-based batteries.

## Methods

### Synthesis of crystalline 2DPI

2DPI was synthesized by the previously reported SMAIS method. Briefly, 50 mL of ultrapure Milli-Q water was firstly injected into a crystallizing dish with a diameter of 50 mm and a height of 60 mm. Then 20 µl surfactant (sodium oleyl sulfate, SOS, 1 mg/ml in chloroform) was spread onto the water surface gently by a microsyringe. After the evaporation of chloroform (~30 min), the solution of 5,10,15,20-Tetrakis(4-aminophenyl)porphyrin (TAPP) monomer (0.7 µmol in triflic acid) was injected into the water phase. A period of 1 h was allowed for the dispersion of TAPP in the water. Next, another monomer, 2,5-dihydroxyterephthalaldehyde (1.4 µmol in water) was injected into the water phase. The reaction was kept at 50 ^o^C for 5 days.

### GIWAXS measurements

The experiments were performed at the XRD1 beamline at Elettra, Trieste, Italy. The detector was a Dectris Pilatus 2 M and the beam energy was 12.399 keV (λ = 1 Å). The sample-detector distance was verified to be 420 mm by using lanthanum hexaboride as a calibration standard. The incidence angle of the beam was chosen to be 0.12° and the exposure time to the beam was 300 s. The resulting images were then analyzed with WxDiff.

### Electrical measurements

The current–voltage (*I–V*) measurements and subsequent energy conversion tests were performed with an electrochemical workstation (CHI). The as-prepared 2DPI membrane was transferred onto a silicon wafer containing an open hole of about 12 μm^2^, and was further mounted between a two-compartment conductivity cell. Standard electrodes (saturated Ag/AgCl salt bridge electrodes, HANA Instruments) were used. KCl was selected as the standard electrolyte because of the close diffusion coefficients of K^+^ and Cl^−^ ions. NaCl electrolyte was selected to imitate the river water and seawater. The electrolyte solutions were adjusted to the desired pH and then injected into each compartment. The testing solutions were all prepared using Milli-Q water (18.2 MΩ cm). The currents are all within the detection limit and no shield method was applied. The current-voltage response was stable during the measurement. For the lifetime testing. The 2DPI membrane was clamped in the electrochemical cell and stayed in the testing solutions all the time, and the testing solutions were replenished before each measurement. The performance was monitored every 12 h.

### Ionic conductance model

In our experiment, the ionic conductance was obtained when there is no concentration gradient on the two sides of the 2DPI membrane. In general, the ionic conductance (*G*) of a nanofluidic channel can be considered as the sum of bulk conductance (*G*_bulk_) and surface conductance (*G*_surface_) as below^30^:

1 $$G={G}_{{{{{{{\rm{bulk}}}}}}}}+{G}_{{{{{{{\rm{surface}}}}}}}}=\left({u}_{+}+{u}_{-}\right)c{N}_{A}{ewd}/l+2{u}_{+}{\sigma }_{s}w/l$$

where ${u}_{+}$ and ${u}_{-}$ are mobility of cation and anion, respectively; *c* is the concentration of bulk solution; *N*_A_ is Avogadro’s number; e is the elementary charge; *d*, *w*, and *l* are the dimensions of the channel, respectively; Here, *d* is the measured thickness of the 2DPI membrane of about 70 nm, and *w* and $l$ are the size of the unit pore of about 2.5 nm. σ_s_ is the surface charge density. *G*_bulk_ dominates at high concentration region, while *G*_surface_ dominates at low concentration region. The number of pores can be deduced from the conductance measured at high concentration of 1 M in which case most of the surface charge will be screened (*G* ≈ *G*_bulk_). Variable surface charge density model was used to fit the experimental values in Fig. 2b. The advantage of variable surface charge model is that it can take the thermodynamic dissociation equilibrium into account. For comparison, values predicted by the constant surface charge density model (12 mC m^−2^) are also plotted.

### Numerical simulation

The experimentally observed ionic diode effect was also investigated using continuum–based 2D Poisson–Nernst–Planck (PNP) model (within COMSOL Multiphysics), which has been validated to be an effective tool to simulate the ion diffusion through nanochannel membranes^35^. In the calculation, the contribution of hydrogen and hydroxyl ions was neglected as their concentrations were much lower than the major ionic carriers in the solution. The fluid was considered to be incompressible. The convective component of current originating from the fluid flow was also not considered. The total ionic flux through a nanochannel, which was contributed by the diffusion current resulting from the concentration gradient and the electrophoretic current induced by potential gradient, can be described by Nernst–Planck equation:

2 $${{{{{{\bf{J}}}}}}}_{{{{{{\bf{i}}}}}}}={D}_{i}\left(\nabla {c}_{i}+\frac{{z}_{i}F{c}_{i}}{{RT}}\nabla \varphi \right)+{c}_{i}{{{{{\bf{u}}}}}}$$

where ***J***_i_ is the ionic flux (*i* = n or p), *D*_*i*_ is the diffusion coefficient, *c*_*i*_ is the ion concentration, *φ* is the electrical potential, *R* is the universal gas constant, *F* is the Faraday constant, *T* is the absolute temperature, ${{{{{\mathbf{u}}}}}}$ is the fluid velocity. The relationship between electrical potential and ion concentration can be described by the Poisson equation:

3 $${\nabla }^{2}\varphi =-\frac{{\rho }_{e}}{{\varepsilon }_{0}{\varepsilon }_{{{\mbox{r}}}}}$$

The dielectric constant of the electrolyte solutions (*ε*) can be calculated as the product of the permittivity of free space (*ε*_0_) and the relative permittivity of the medium (*ε*_r_). The space charge density (*ρ*_e_) can be given as:

4 $${\rho }_{e}=F{\sum }_{k=1}^{m}{z}_{i}{c}_{i}$$

where m was the number of ionic species. The ionic flux for each ions should satisfy the continuity equation when the system reached stationary regime:

5 $$\nabla \cdot {{{{{{\bf{J}}}}}}}_{{{{{{\bf{i}}}}}}}=0$$

In order to gain an affordable commutation scale, the fluidic transport route was simplified to be 1000 nm. Two large electrolyte reservoirs were added to reduce the influence of the resistance of mass transfer at the entrance and exit. The electrostatic boundary condition for potential *φ* on the channel wall is

6 $${{{{{\bf{n}}}}}}\cdot{{{{{\boldsymbol{\cdot }}}}}}\nabla \varphi =-\frac{\sigma }{{\varepsilon }_{0}{\varepsilon }_{r}}$$

where *σ* is the surface charge density. Impermeable boundary conditions were employed at both the entrance for ionic concentration and electric field. The ionic flux had the zero normal components at boundaries:

7 $${{{{{\bf{n}}}}}}\cdot {{{{{\bf{J}}}}}}_{{{{{\bf{i}}}}}}=0$$

The simulation model and detailed parameters are shown in Supplementary Figs. 14, 15 and Supplementary Table 3, respectively. By solving the coupled Eqs. (2)–(5) with proper boundary conditions (6)–(7), the ion concentration distributions inside the nanochannel can be obtained.

### Ion selectivity coefficient

Under a concentration gradient, the recorded osmotic potential (*V*_os_) originates from the difference in the diffusive fluxes of cations and anions. *V*_os_ can be described as^41^

8 $${V}_{{{{{{{\rm{os}}}}}}}}=S\frac{{RT}}{F}\triangle {{{{{\rm{ln}}}}}}C$$

where *R*, *T*, and *F* are the universal gas constant, the absolute temperature, and the Faraday constant, respectively; ΔIn*C* is the natural logarithm of the salinity ratio between concentrated and diluted sides; *S* is the ion selectivity coefficient that is defined as *t*_+_ – *t*_–_ (*t*_+_ and *t*_–_ are the ion transference number of cation and anion, respectively). For cation-selective membranes, *S* ranges from 0 to 1 where 0 refers to non-selective case and 1 refers to ideally ion-selective case.

The Na^+^/Cl^‒^ and K^+^/Cl^‒^ selectivity ratio (SR) can be obtained^42^:

9 $${{{{{{\rm{SR}}}}}}}=\frac{{t}_{+}}{1-{t}_{+}}$$

### Computational methodology

Full structural optimization (relaxation of lattice vectors and atomic positions) to obtain the most stable periodic model of 2DPI are performed using SCC-DFTB^45^ as implemented in the Amsterdam Density Functional (ADF)–DFTB 2019 program suite (www.scm.com). The 3ob-3-1^46^ set for the *X*–*Y* element pair interaction (*X*, *Y* = C, H, N, O) is employed. The 2D bilayer model is used to analyze various stacking possibility within the two possible configurations of stacking, i.e., in-phase and out-of-phase configurations, resultant of the collective rotation of the TAPP units (Supplementary Fig. 7). The two-layered unit cell bulk structure is simulated from the most stable 2D bilayer model (model 13 in Supplementary Fig. 7).

Further calculations with the periodic bulk structures are performed using the Vienna ab initio simulation package (VASP version 5.4.4)^47,48^. The projector augmented wave (PAW) method combined with the Perdew–Burke–Ernzerhof (PBE) generalized gradient approximation is applied for the exchange-correlation potential^49–52^. The DFT-D3 extension of Grimme is adopted for the correction of van der Waals interactions that adds the vdW correction for potential energy and interatomic forces^53^. In all calculations, a convergence threshold of 10^−3^ eV in energy and 10^−2^ eV Å^−1^ for the force with a cutoff energy of 470 eV for the plane-wave basis set are adopted. The Brillouin zone is sampled with a Gamma centered k-point grid of 2 × 2 × 6 for bulk calculations. The climbing image nudged elastic band (CI-NEB) method is used to locate the diffusion pathway^54,55^. The minimum energy paths (MEP) are initialized by considering seven image structures between initial and final structural geometries, and the energy convergence criterion of each image is set to 10^−3^ eV. Activation barriers are calculated by the energy differences between the transition and initial states.

The binding energy (*E*_binding_) is calculated by subtracting the total energy of the metal-ion hydrates bound to the 2DPI structure (${E}_{{{{{{\rm{P}}}}}}+{{{{{\rm{M}}}}}}{\cdot}n{{{{{{\rm{H}}}}}}}_{2}{{{{{\rm{O}}}}}}}$) from the sum of the energies of the relaxed bare 2DPI structure (*E*_P_), the gas phase of metal (*E*_M_) and the corresponding isolated water molecules in the gas phase (${E}_{{{{{{{\rm{H}}}}}}}_{2}{{{{{\rm{O}}}}}}}$**)**:

10 $${E}_{{{{{{{\rm{binding}}}}}}}}={E}_{{{{{{\rm{P}}}}}}+{{{{{\rm{M}}}}}}.n{{{{{{\rm{H}}}}}}}_{2}{{{{{\rm{O}}}}}}}-{E}_{{{{{{\rm{M}}}}}}}-{E}_{{{{{{\rm{P}}}}}}}-6{E}_{{{{{{{\rm{H}}}}}}}_{2}{{{{{\rm{O}}}}}}}$$

## Supplementary information

Supplementary Information

## Data Availability

The data that supports the findings of this study are available from the corresponding author upon request.
