# Supplementary material for: Cation-selective two-dimensional polyimine membranes for high-performance osmotic energy conversion
Source: Nat Commun. 2022 Jul 8;13:3935. doi: 10.1038/s41467-022-31523-w (PMC9270359; doi:10.1038/s41467-022-31523-w)
Supplement: Supplementary file 1 — Supplementary Information [file 41467_2022_31523_MOESM1_ESM.pdf]

Supplementary Information

for

Cation-Selective Two-Dimensional Polyimine Membranes for High-Performance Osmotic Energy Conversion

Zhen Zhang[1,2,3,4,8], Preeti Bhauriyal[1,8], Hafeesudeen Sahabudeen[1], Zhiyong Wang[1,2], Xiaohui Liu[1], Mike Hambsch[5], Stefan C. B. Mannsfeld[5], Renhao Dong[1*], Thomas Heine[1,6,7*] and Xinliang Feng[1,2*]

[1]Center for Advancing Electronics Dresden (cfaed) and Faculty of Chemistry and Food Chemistry, Technische Universität Dresden, 01062 Dresden, Germany
[2]Max Planck Institute of Microstructure Physics, Halle (Saale) 06120, Germany
[3]Suzhou Institute for Advanced Research, University of Science and Technology of China, Suzhou, Jiangsu 215123, China
[4]School of Chemistry and Materials Science, University of Science and Technology of China, Hefei 230026, China
[5]Center for Advancing Electronics Dresden (cfaed) and Faculty of Electrical and Computer Engineering, Technische Universität Dresden, 01062 Dresden, Germany
[6]Helmholtz Center Dresden-Rossendorf, Institute of Resource Ecology, Leipzig Research Branch, Permoserstr. 15, 0416 Leipzig, Germany
[7]Department of Chemistry, Yonsei University, Seodaemun-gu, Seoul 120-749, Republic of Korea
[8]These authors contributed equally
[*]Corresponding author: renhao.dong@tu-dresden.de; thomas.heine@tu-dresden.de; xinliang.feng@tu-dresden.de

This PDF file includes:

Materials and Methods

Supplementary Figures 1 to 33

Supplementary Tables 1 to 3

Supplementary References 1-18

Materials and Methods

Chemicals: Trifluoromethanesulfonic acid and 5,10,15,20-Tetrakis(4-aminophenyl)porphyrin (TAPP) were purchased from TCI. 2,5-dihydroxyterephthalaldehyde (DhTPA) was purchased from ChemPur. Sodium oleyl sulfate was purchased from Sigma-Aldrich. The electrolyte potassium chloride (KCl), sodium chloride (NaCl), calcium chloride ($CaCl_2$), magnesium chloride ($MgCl_2$), aluminium chloride ($AlCl_3$), hydrochloric acid (HCl), and potassium hydroxide (KOH) were all purchased from Sigma-Aldrich. Ultrapure water (18.2 $M\Omega \cdot cm^{-1}$) was used for preparing testing ionic solutions.

Characterization: The scanning electron microscopy (SEM) was performed using Gemini 500 (Zeiss, Germany). Atomic force microscopy (AFM) was performed using Multimode-8 (Bruker, USA). The X-ray photoelectron spectroscopy (XPS) analysis was carried out using ESCALAB 250Xi. Ultraviolet-visible spectroscopy was carried out on Cary 5000 UV-vis-NIR spectrophotometer (Agilent). Transmission electron microscopy (TEM) image was captured with Libra200 (Zeiss) at an accelerating voltage of 200 kV. Zeta potential of membrane surface was measured using SurPASS™ 3 Zeta Potential Analyzer (Anton Paar). Raman spectra were collected on a Renishaw inVia Raman microscope.

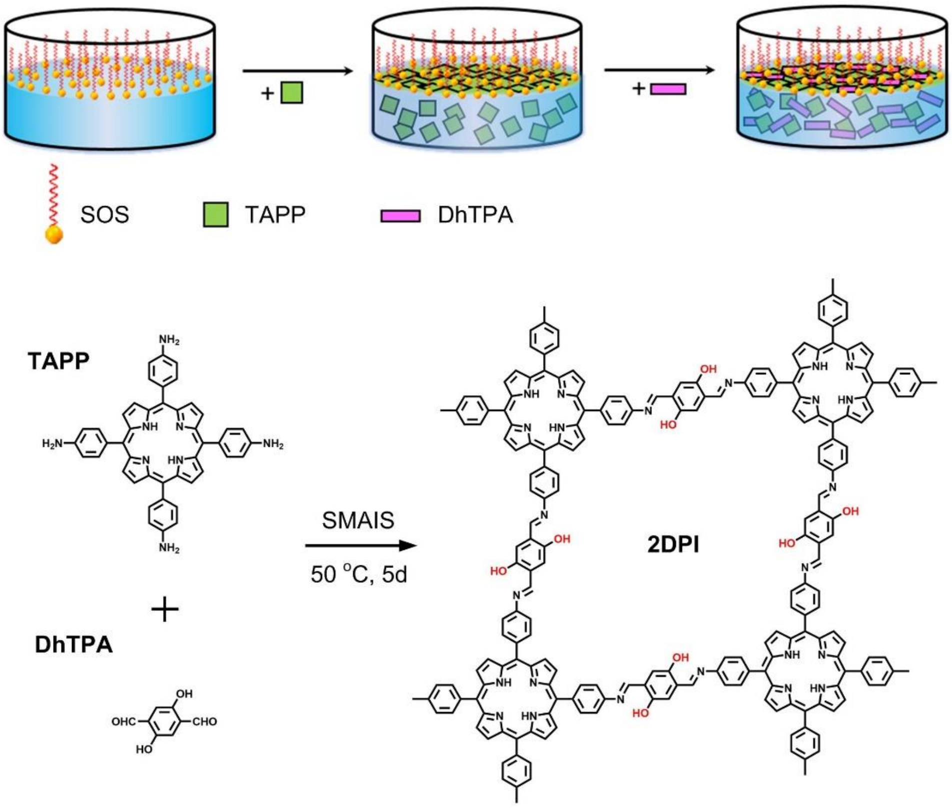

Supplementary Figure 1. Schematic of the surfactant-monolayer-assisted interfacial synthesis (SMAIS) method (top), and the corresponding Schiff-base condensation reaction between TAPP and DhTPA (bottom).

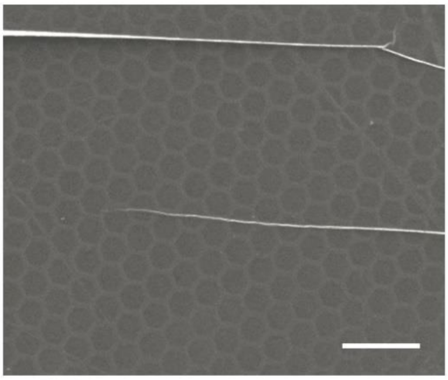

Supplementary Figure 2. SEM image of the as-prepared 2DPI membrane supported on copper mesh grid (scale bar: 100 µm).

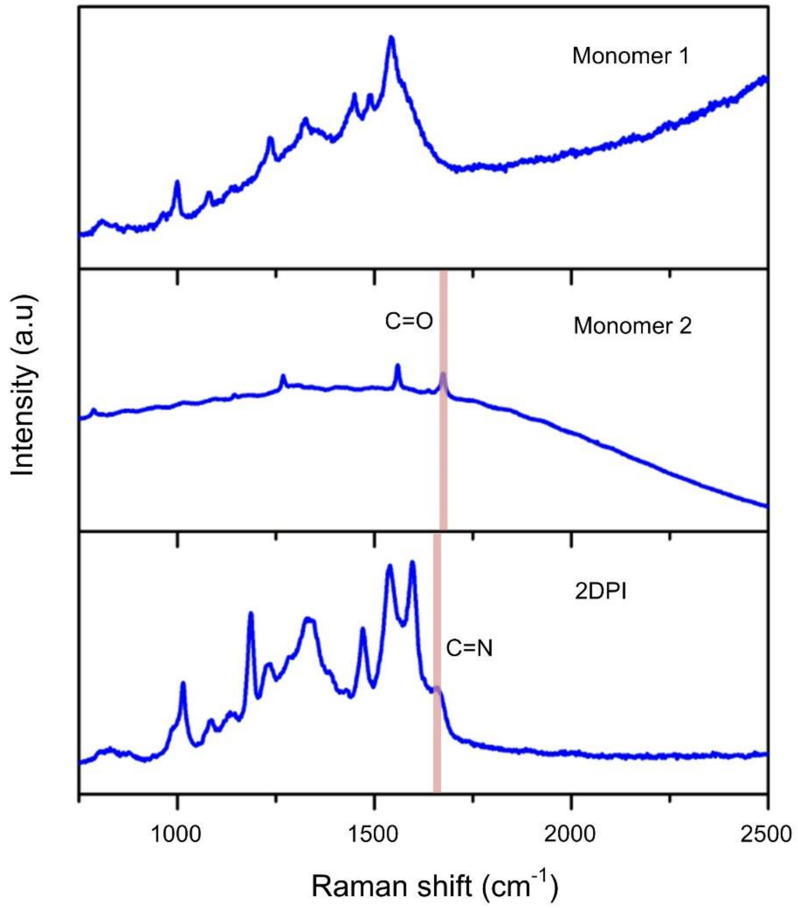

Supplementary Figure 3. Raman of monomer 1 (TAPP), monomer 2 (DhTPA), and 2DPI membrane. For the 2DPI membrane, the characteristic aldehyde C=O stretch peak of DhTPA at 1675 cm^{-1} disappears and a new peak of C=N at 1659 cm^{-1} emerges, indicating that aldehyde monomers were efficiently transformed into imine polymers.

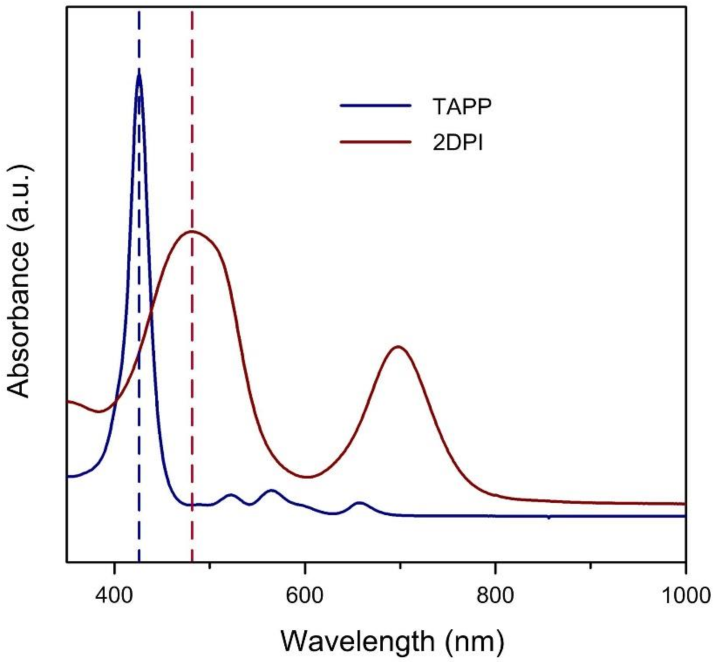

Supplementary Figure 4. UV-vis spectra of TAPP monomer and 2DPI membrane. Compared with TAPP, the Soret band (dashed line) of 2DPI membrane red-shifts by about 55 nm, which can be ascribed to its extended conjugation system.

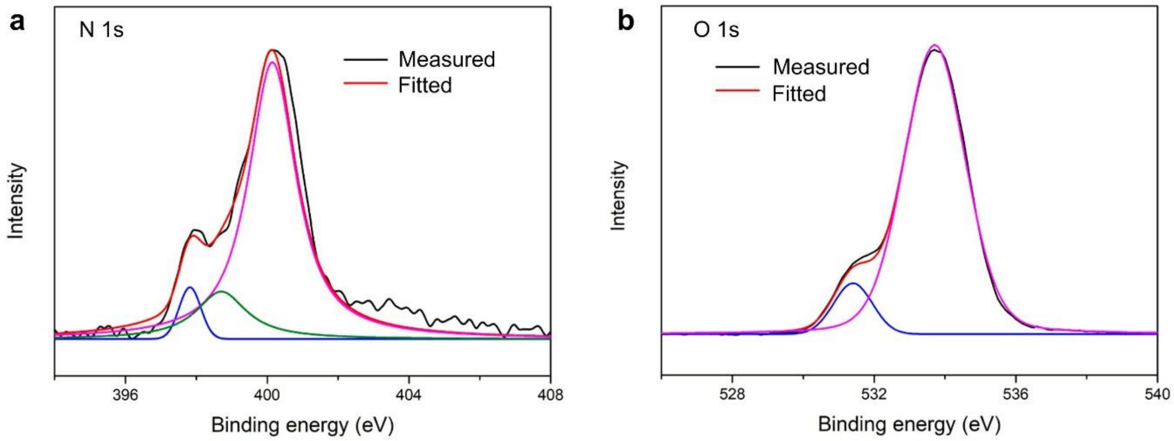

Supplementary Figure 5. XPS spectra of the 2DPI membrane. (a) N 1s: The two peaks at 397.8 eV and 400.1 eV can be assigned to the two different types of N atom of the porphyrin structure in 2DPI membrane, while the peak at 398.7 eV can be ascribed to the linking imine bond.[1, 2] (b) O 1s: The existence of phenol groups evidenced by a strong peak at 533.7 eV.[3, 4]

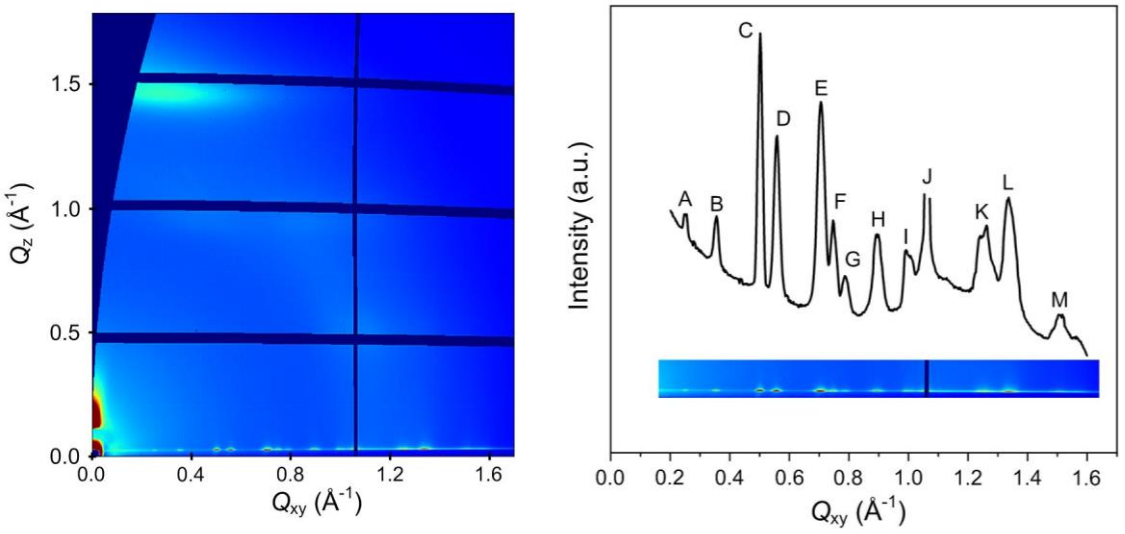

	hkl	Qxy (Å^{-1})
A	(100)	0.252
B	(110)	0.355
C	(200)	0.502
D	(210)	0.558
E	(220)	0.706
F	(300)	0.748
G	(130)	0.788
H	(320)	0.895
I	(400)	0.995
J	(330)	1.06
K	(340)	1.25
L	(520)	1.34
M	(600)	1.509

Supplementary Figure 6. GIWAXS pattern of the 2DPI membrane, indicating its excellent crystallinity over the macroscale.

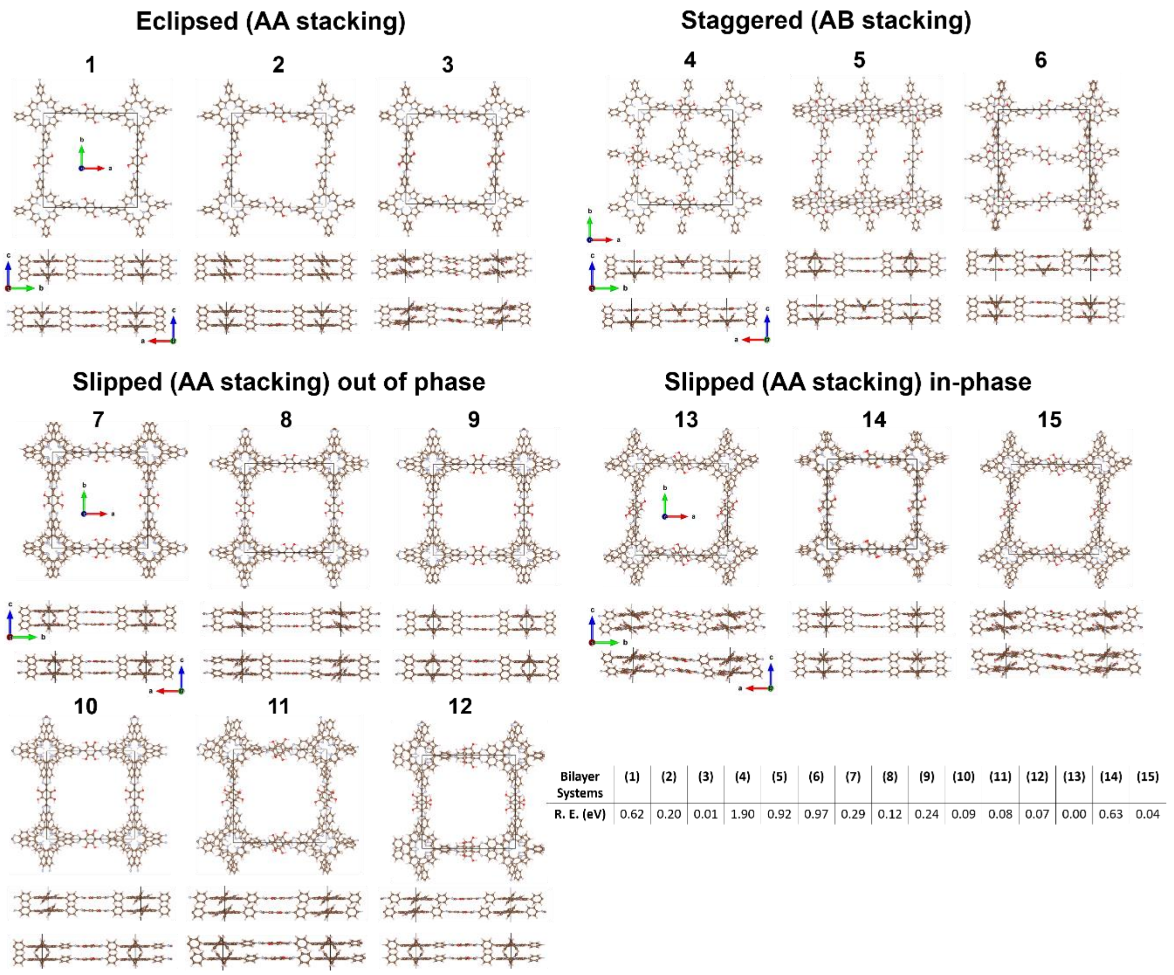

Bilayer Systems	(1)	(2)	(3)	(4)	(5)	(6)	(7)	(8)	(9)	(10)	(11)	(12)	(13)	(14)	(15)
R. E. (eV)	0.62	0.20	0.01	1.90	0.92	0.97	0.29	0.12	0.24	0.09	0.08	0.07	0.00	0.63	0.04

Supplementary Figure 7. (1-15) DFTB models of various stacking possibilities for 2DPI bilayer and the corresponding relative energies (R.E.) obtained using DFTB. Here, red, pink, brown and blue color represent oxygen, hydrogen, carbon, and nitrogen atoms, respectively.

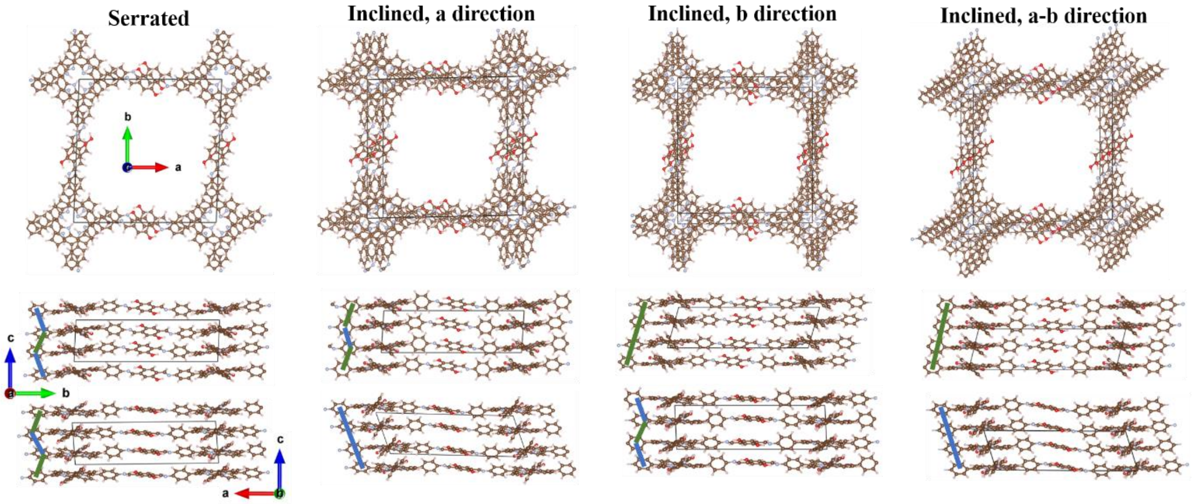

	a	b	c	α	β	γ	R.E. (eV)
Serrated	25.54	25.60	7.44	87.30	86.22	87.58	1.37
a-direction	25.64	25.46	8.17	86.23	69.34	88.85	0.36
b-direction	25.46	25.66	7.74	74.43	87.23	89.50	0.76
a-b-direction	25.49	25.59	7.69	76.32	72.34	87.77	0.00

Supplementary Figure 8. The modelled AA-stacked in-phase configurations of bi-layered bulk structures. The corresponding lattice parameters and relative energies (R.E.) obtained using DFTB.

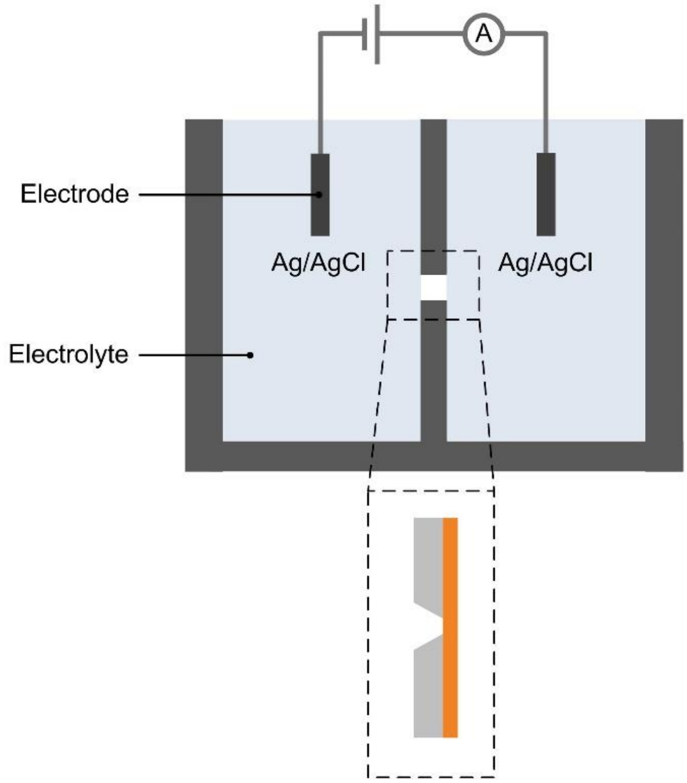

Supplementary Figure 9. Schematic of the setup for electric measurement.

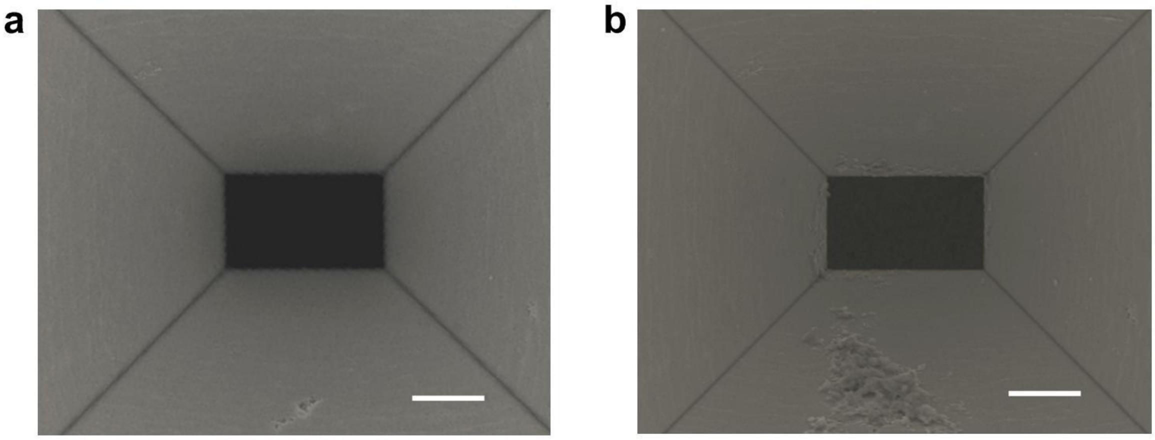

Supplementary Figure 10. SEM images of the silicon wafer with a single hole of about 11.8 um^2 before (a) and after (b) transfer of the 2DPI membrane (scale bar, 1 μm).

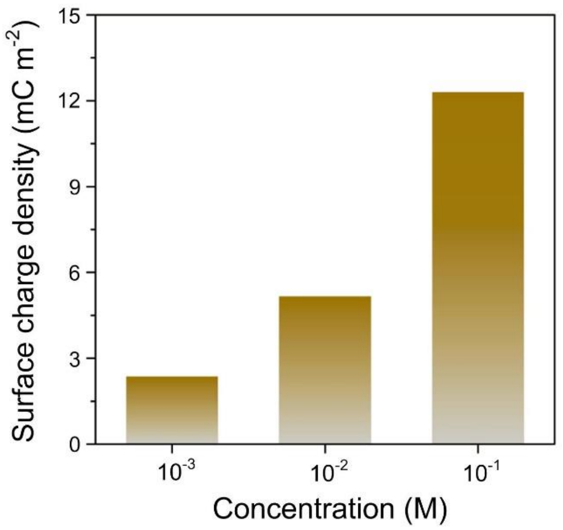

Supplementary Figure 11. Surface charge density as a function of KCl concentration by fitting the conductance data with variable charge model.

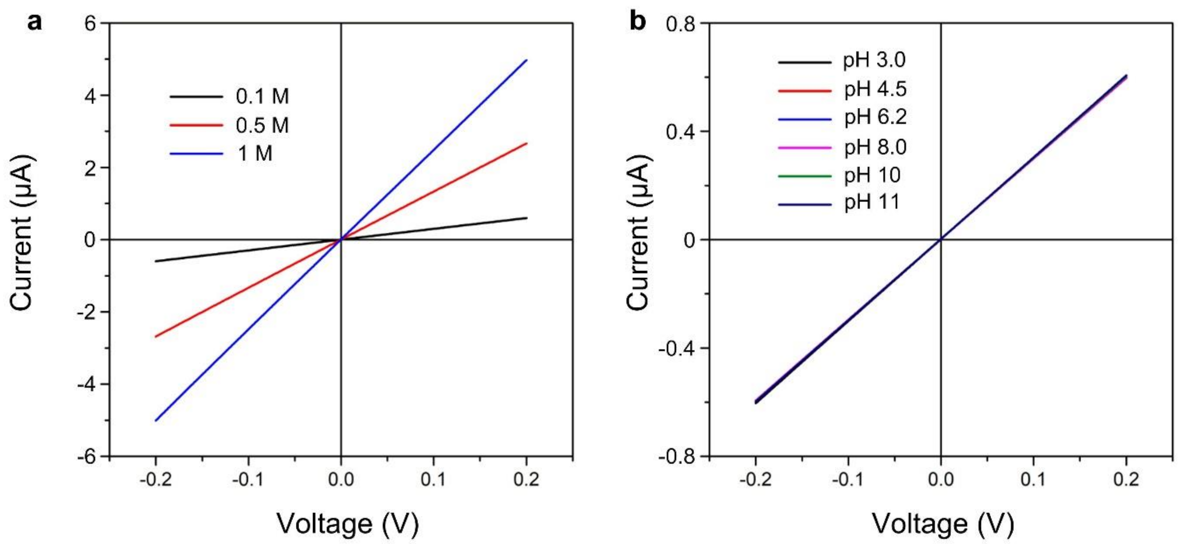

Supplementary Figure 12. (a) *I*-*V* curves of the blank silicon hole recorded in KCl electrolyte solutions of different concentrations. (b) *I*-*V* curves of the blank silicon hole recorded in 0.1 M KCl electrolyte solutions of different pH values.

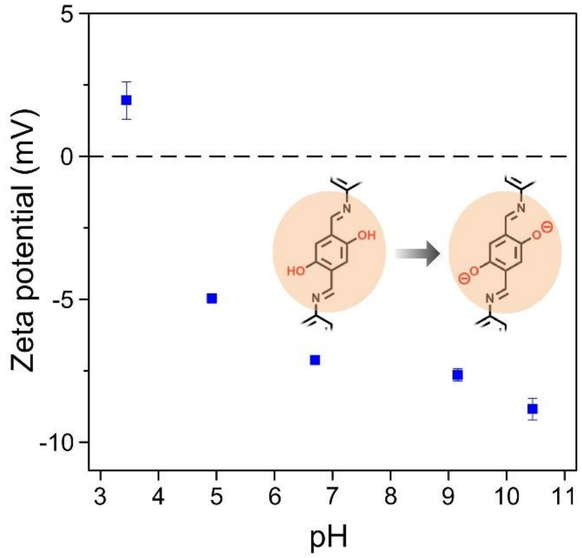

Supplementary Figure 13. Surface Zeta potential measurement of the 2DPI membrane at pH from 3 to 11.

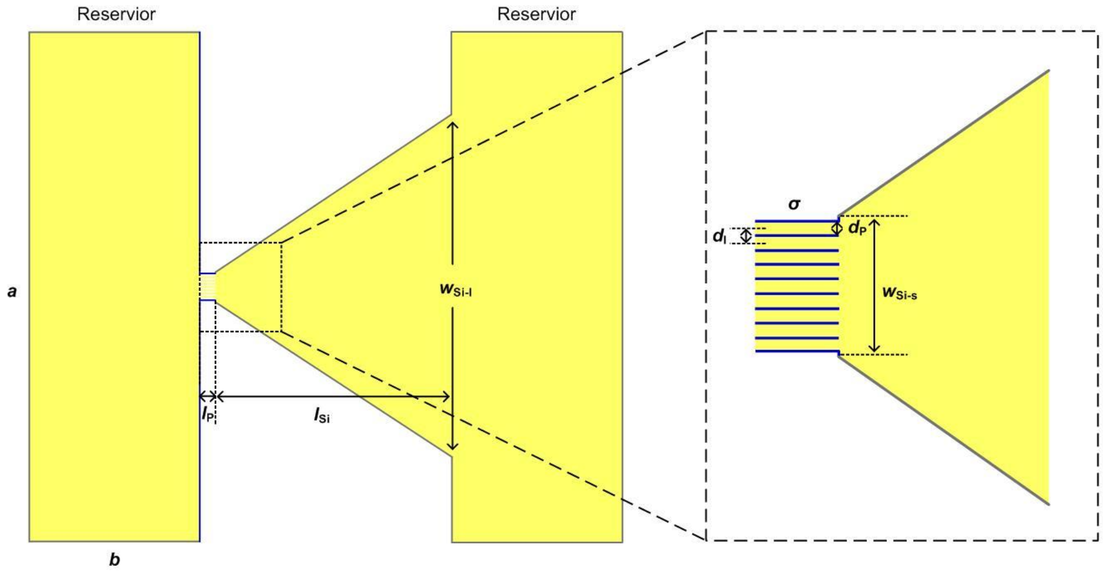

Supplementary Figure 14. Numerical simulation model which contains about 1100 2.32 nm-wide 2DP channels connected by one 3000 nm-wide silicon conical channel (drawing not to scale).

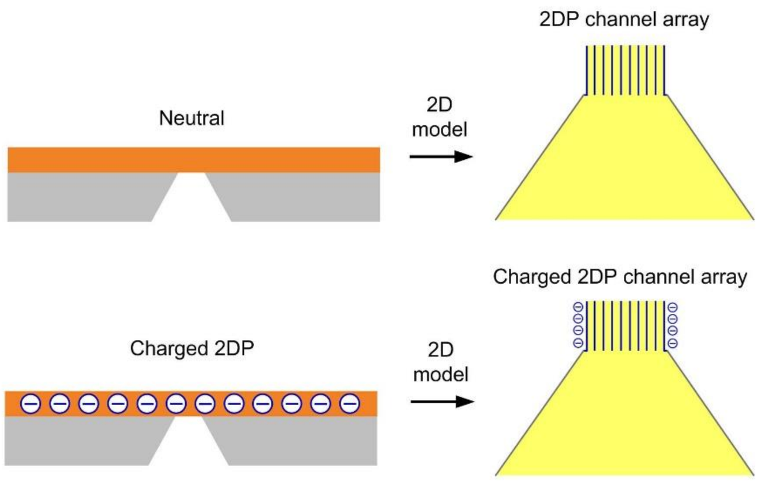

Supplementary Figure 15. Two theoretical models simulating the neutral 2DP coated substrate and charged 2DP coated substrate (drawing not to scale).

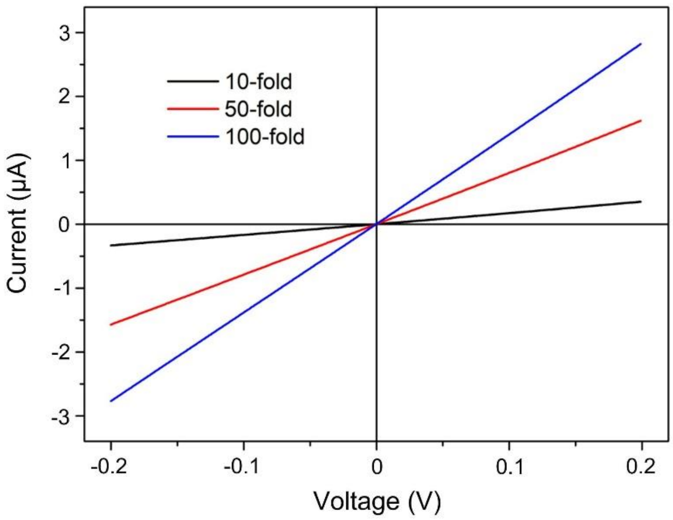

Supplementary Figure 16. *I-V* curves of the blank silicon hole recorded in KCl electrolyte solutions of different concentration gradient. The low concentration side is fixed at 0.01 M.

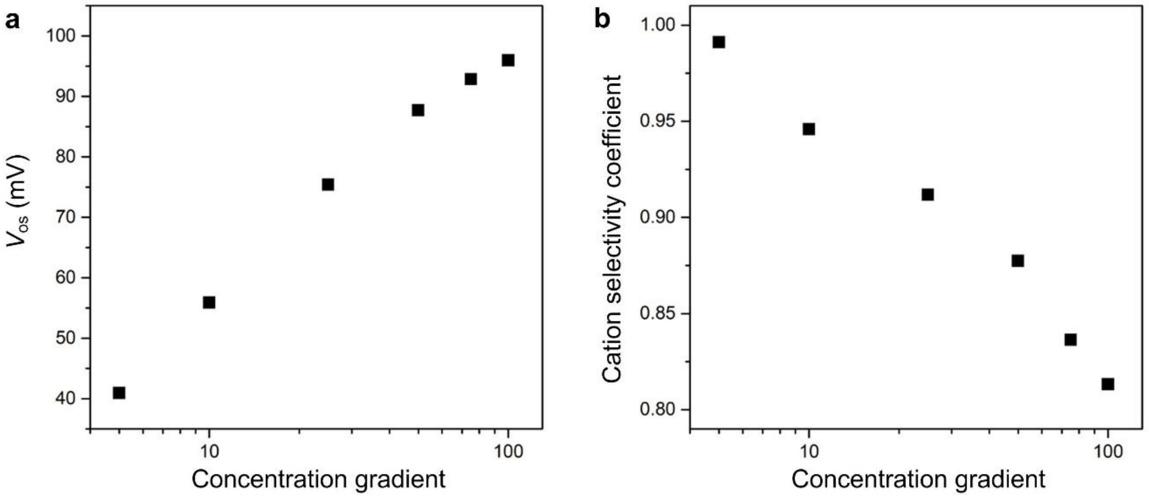

Supplementary Figure 17. Measured V_{os} (a) and cation selectivity coefficient (b) of the sub-micrometer-scale device. The low concentration side is fixed at 0.01 M KCl.

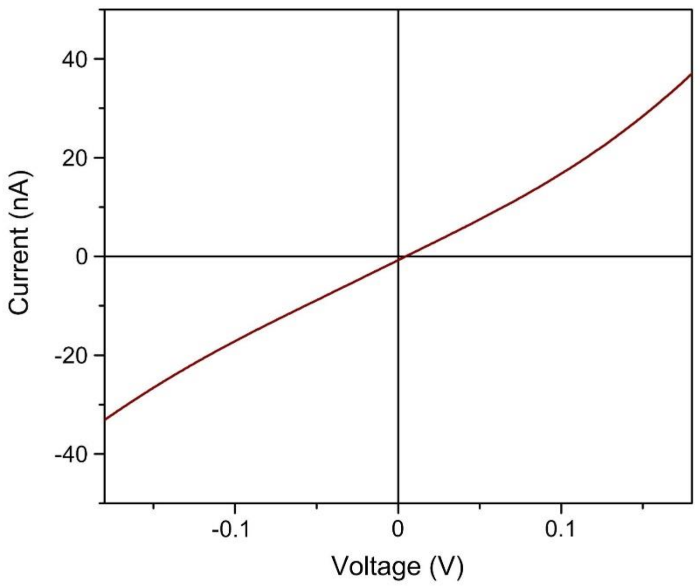

Supplementary Figure 18. *I*-*V* curve of the 2DPI membrane recorded at pH 3 under 10-fold KCl concentration gradient (0.1/0.01 M).

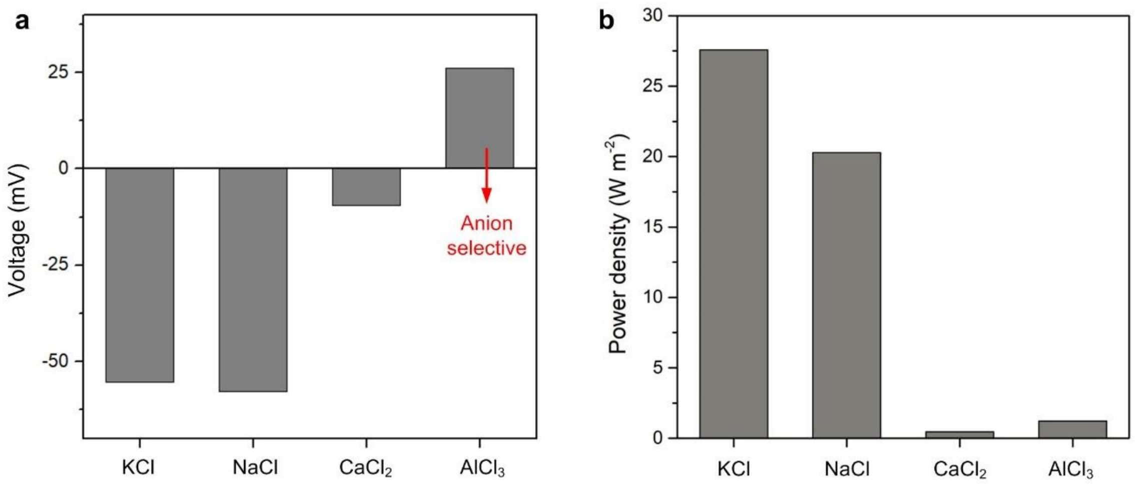

Supplementary Figure 19. Measured osmotic potential (a) and power density (b) of the 2DPI membrane as a function of electrolyte species (the concentration gradient is 10-fold, 0.1/0.01 M).

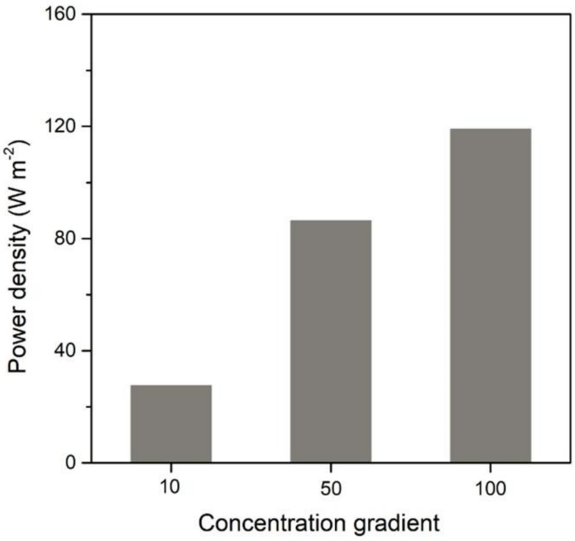

Supplementary Figure 20. Theoretical power density of 2DPI membrane under a series of KCl concentration gradient.

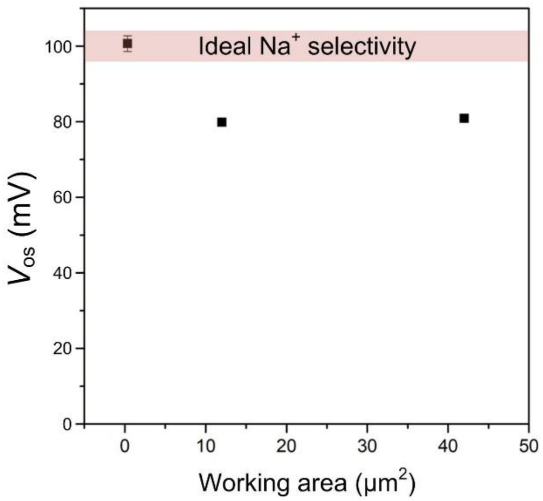

Supplementary Figure 21. V_{os} of the 2DPI membrane with different working area under 50-fold NaCl concentration gradient. The sub-micrometer-scale device can realize ideal cation selectivity ~1.

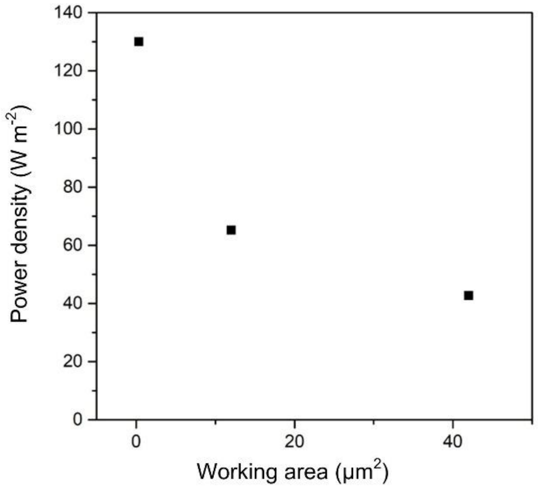

Supplementary Figure 22. Power density of the 2DPI membrane with different working area.

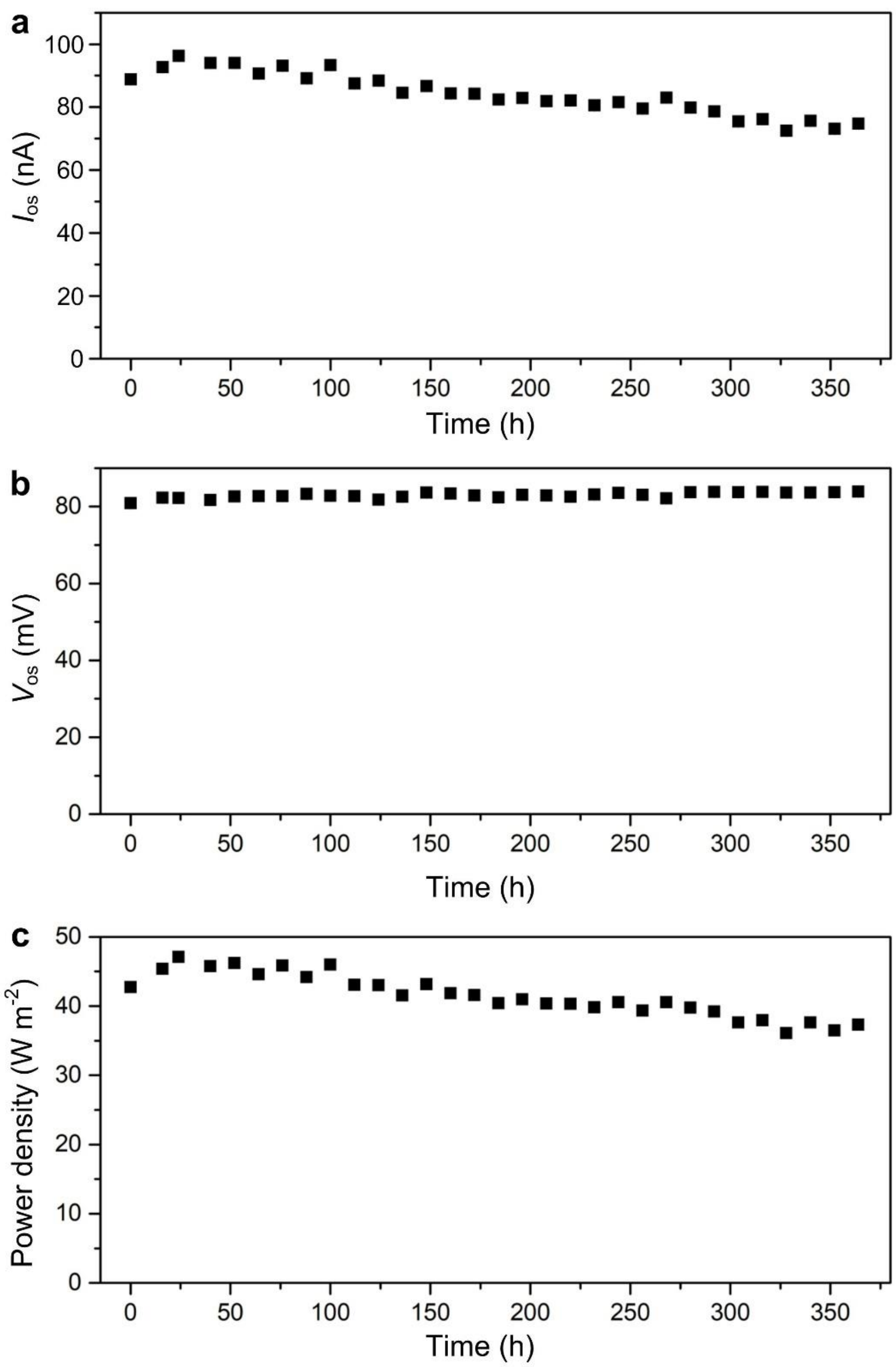

Supplementary Figure 23. I_{os} (a), V_{os} (b), and power density (c) of 2DPI membrane recorded under 0.5/0.01 M NaCl concentration gradient with increasing testing time.

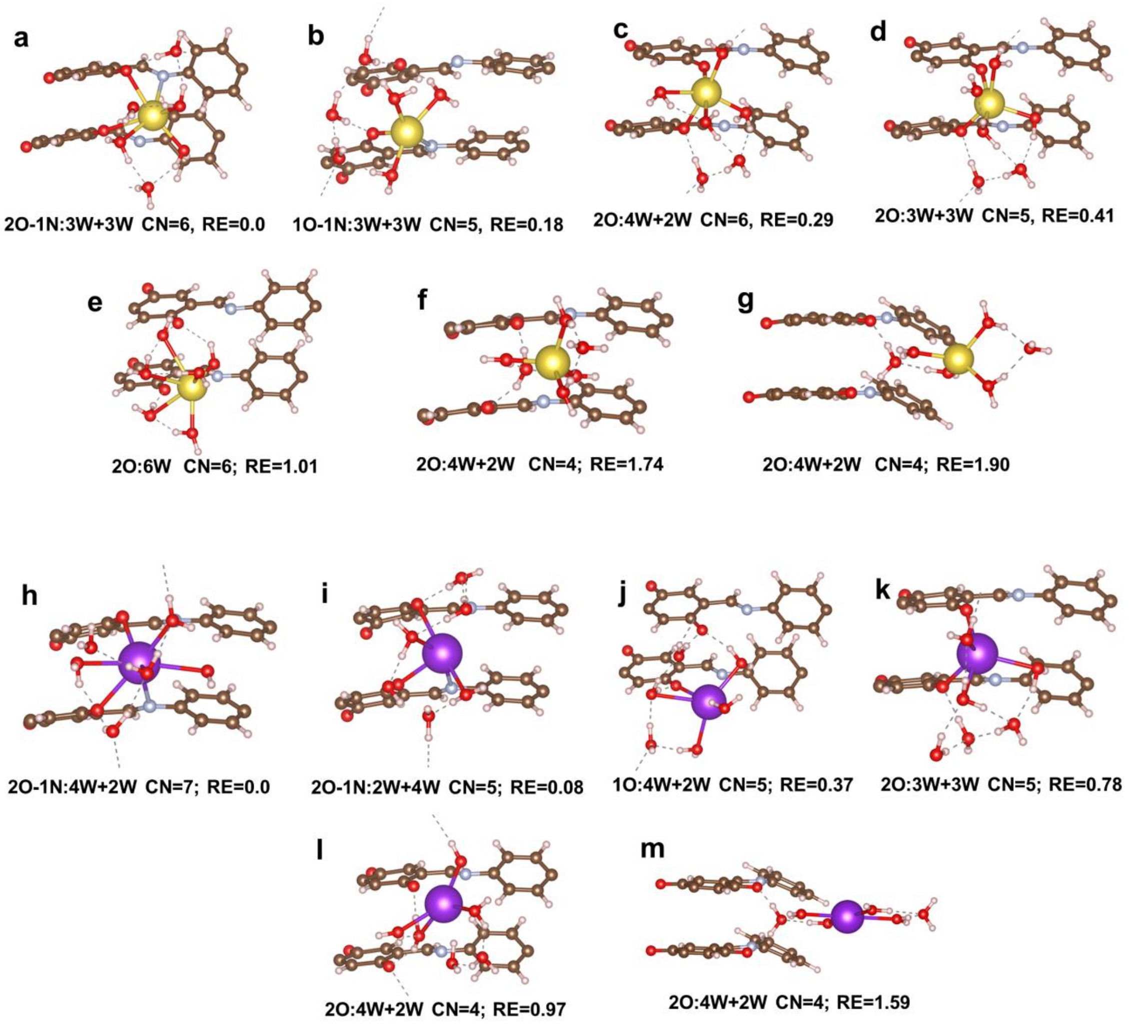

Supplementary Figure 24. Side views of stable interaction sites of $Na(H_2O)_6$ (a-d) direct binding, (e-g) indirect binding; and of $K(H_2O)_6$ system (h-k) direct binding, and (i-m) indirect binding with fully deprotonated polymer. Here, mW+nW represents, m, n number of water molecules in first and second hydration shell, respectively, CN is the coordination number of Na^+/K^+ ion and RE is the relative energy difference between the sites in eV.

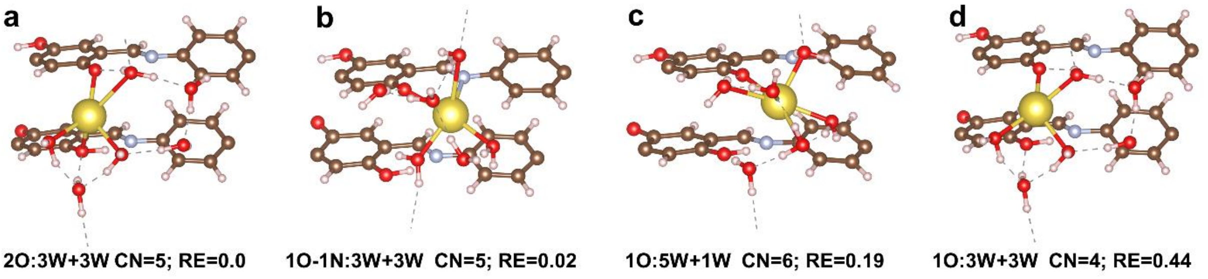

Supplementary Figure 25. (a-d) Side views of stable interaction sites of $Na(H_2O)_6$ with partially deprotonated polymer. Here, mW+nW represents, m, n number of water molecules in the first and second hydration shell, respectively, CN is the coordination number of Na^+/K^+ ion and RE is the relative energy difference between the sites in eV.

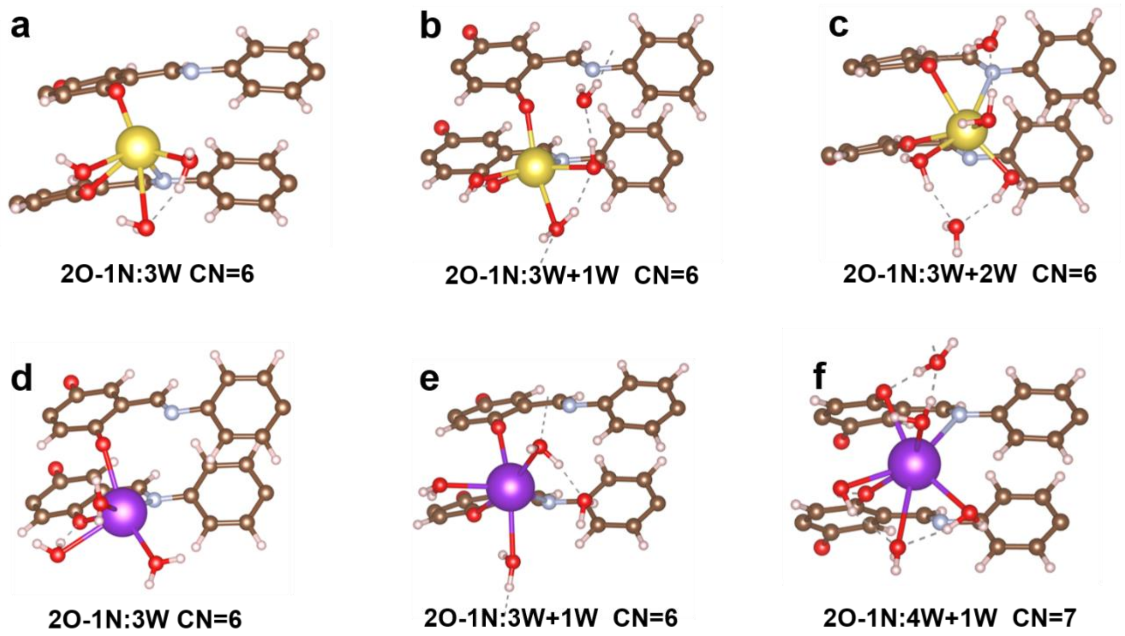

Supplementary Figure 26. Side views of most stable interaction sites of (a-c) $Na(H_2O)_x$ and (d-f) $K(H_2O)_x$ with fully deprotonated polymer, where, x=3, 4, and 5. Here, mW+nW represents, m, n number of water molecules in the first and second hydration shell, respectively, CN is the coordination number of Na^+/K^+ ion.

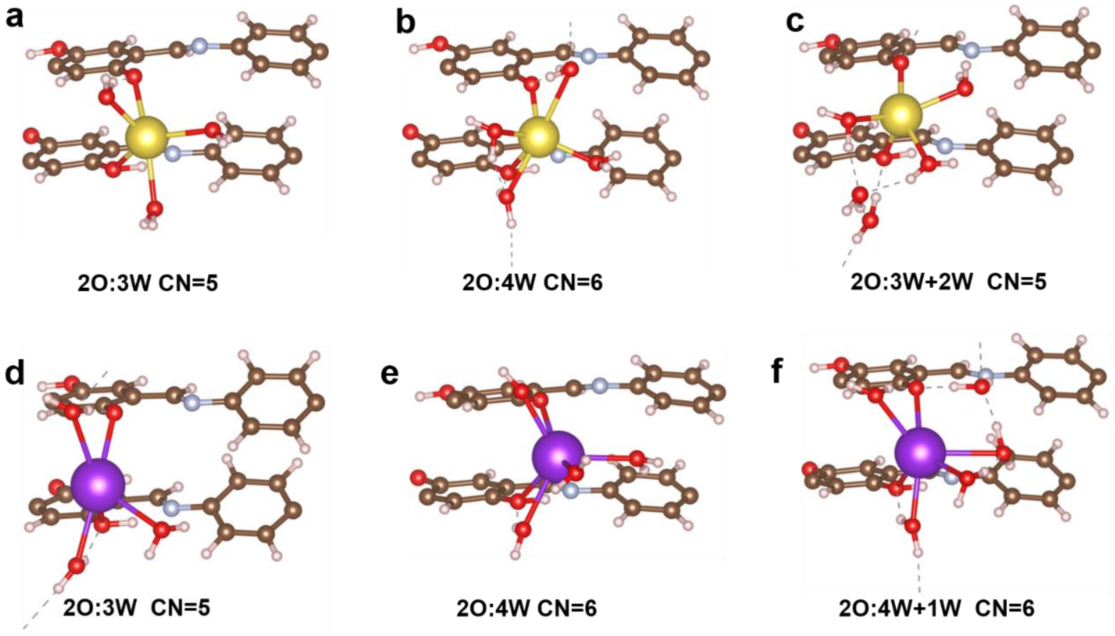

Supplementary Figure 27. Side views of the most stable interaction sites of (a-c) $Na(H_2O)_x$ and (d-f) $K(H_2O)_x$ with partially deprotonated polymer, where, x=3, 4, and 5. Here, mW+nW represents, m, n number of water molecules in the first and second hydration shell, respectively, CN is the coordination number of Na^+/K^+ ion.

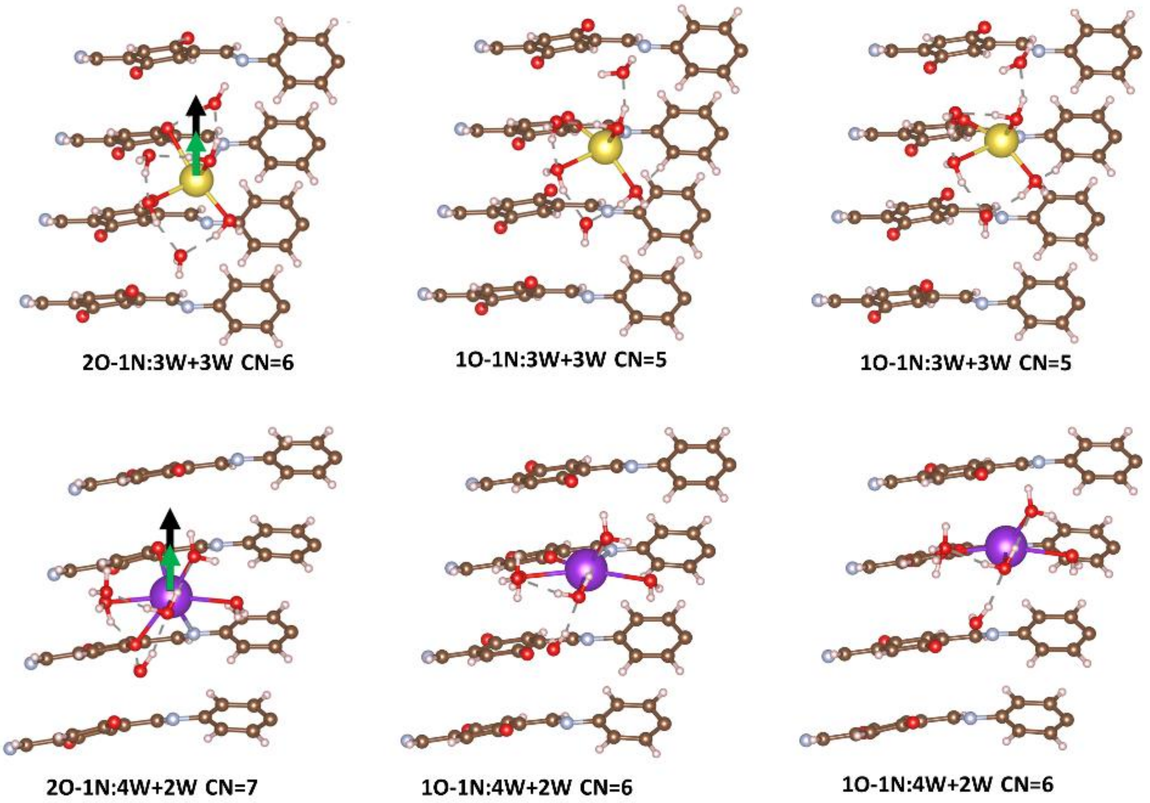

Supplementary Figure 28. Detailed diffusion pathways for $Na(H_2O)_6$ and $K(H_2O)_6$ on fully deprotonated 2DPI surface. The images (from left to right) represent the initial, transition and final state of diffusion path, respectively.

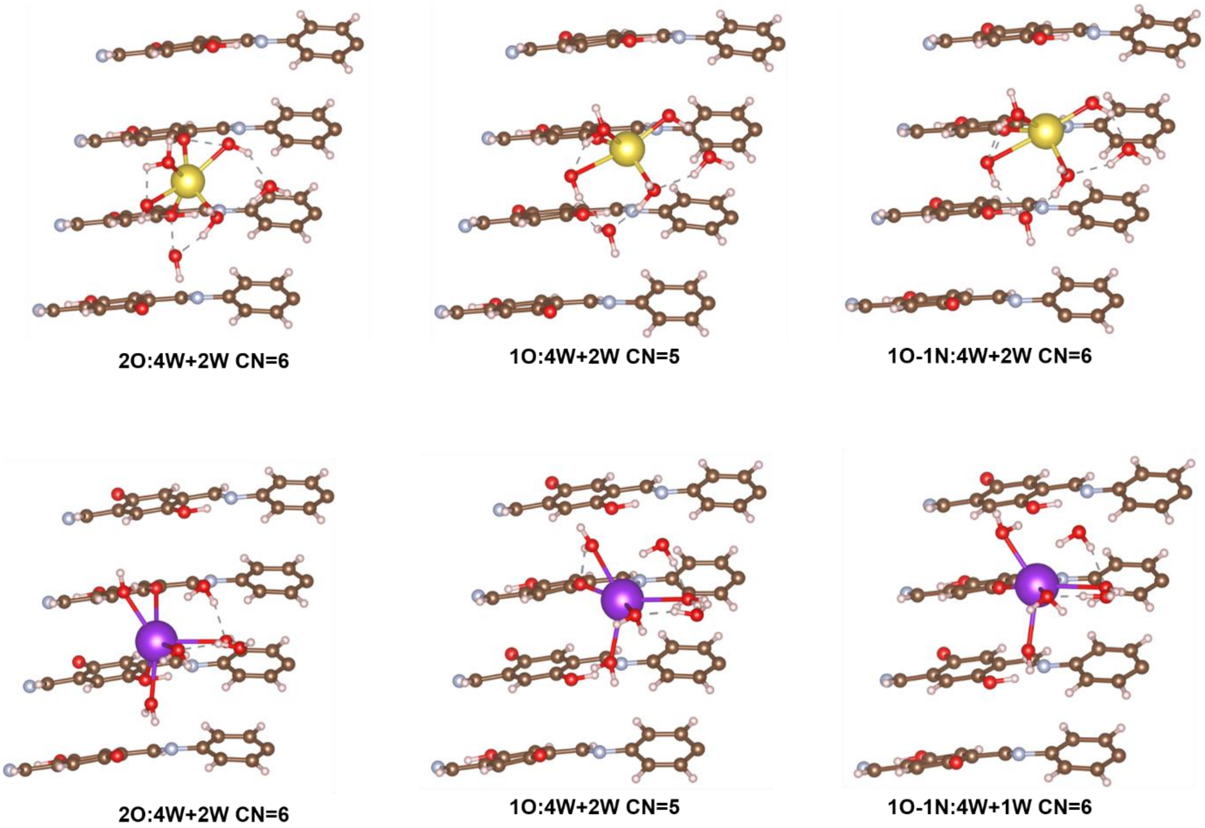

Supplementary Figure 29. Detailed diffusion pathways for $Na(H_2O)_6$ and $K(H_2O)_6$ on partially deprotonated 2DPI surface. The images (from left to right) represent the initial, transition and final state of diffusion path, respectively.

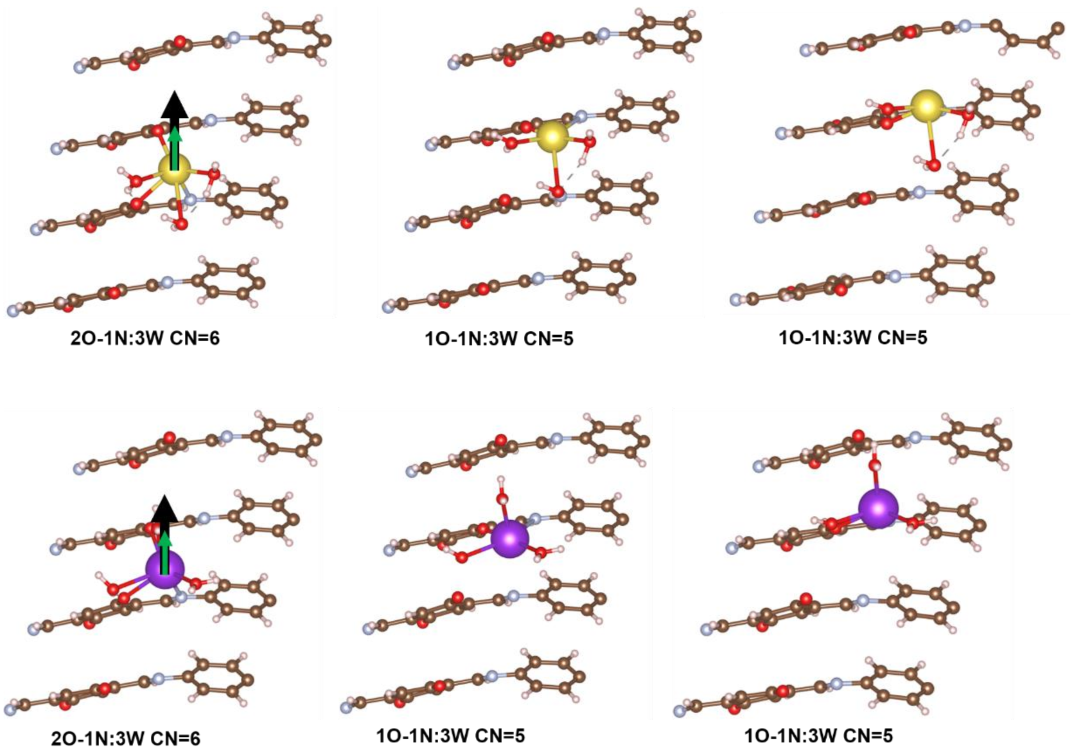

Supplementary Figure 30. Detailed diffusion pathways for $Na(H_2O)_3$ and $K(H_2O)_3$ on fully deprotonated 2DPI surface. The images (from left to right) represent the initial, transition and final state of diffusion path, respectively.

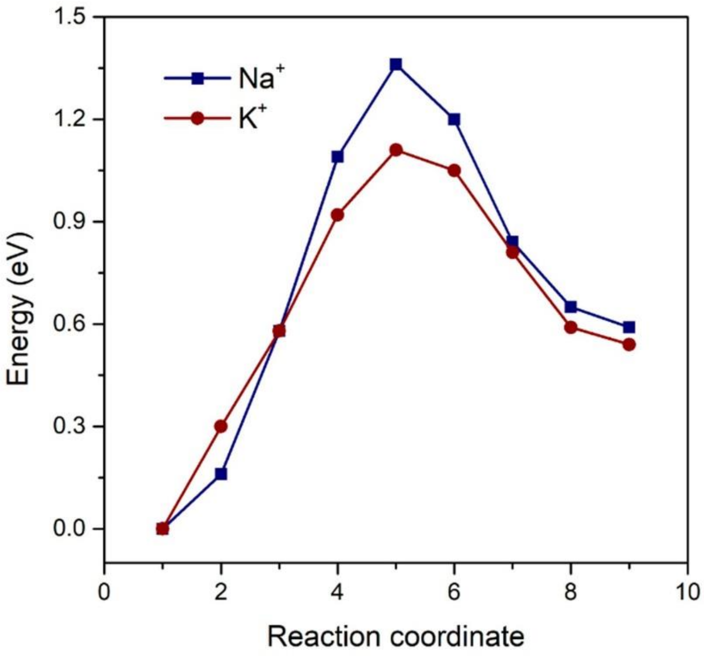

Supplementary Figure 31. Diffusion energy profile for $Na(H_2O)_3$ and $K(H_2O)_3$ on fully deprotonated 2DPI surface.

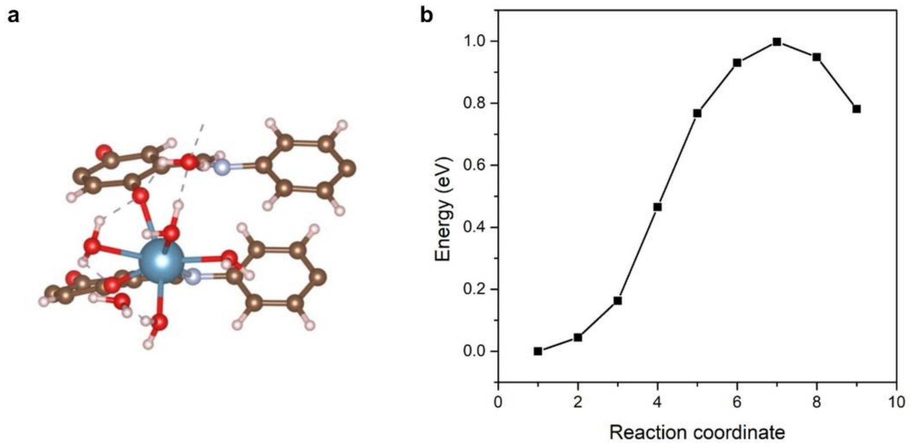

Supplementary Figure 32. (a) The most stable binding site of $Ca(H_2O)_6$ on fully deprotonated 2DPI surface (2O-1N binding site). (b) Energy profile of diffusion path of $Ca(H_2O)_6$.

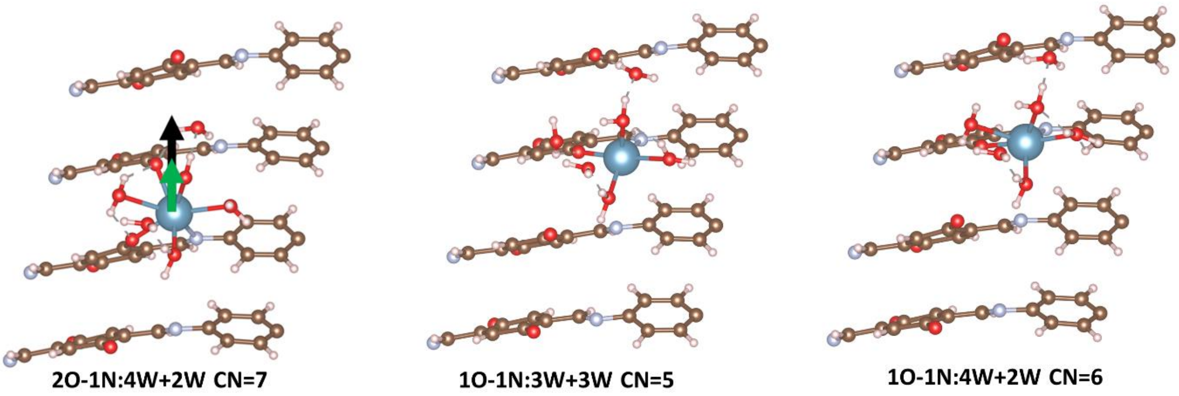

Supplementary Figure 33. Detailed diffusion pathways for $Ca(H_2O)_6$ on fully deprotonated 2DPI surface. The images (from left to right) represent the initial, transition and final state of diffusion path, respectively.

Supplementary Table 1. Comparison of potassium and sodium ion selectivity.

Type	Concentration gradient (M)	Ion transference number	Ion type	Selectivity ratio	Ref
Aromatic hydrocarbon membrane	–	0.7	K^+	–	5
Angstrom-scale slit	0.1: 0.01	–	K^+	3	6
GO membrane	0.01: 10^{-5}	0.85	K^+	–	7
Vertical GO membrane	1: 0.001	0.916	Na^+	–	8
GO/SNF membrane	–	0.7-0.8	K^+	–	9
BP membrane	1: 0.01	0.83	K^+	–	10
MXene/ANF membrane	0.025: 10^{-4}	0.8	K^+	–	11
MXene membrane	0.1: 10^{-4}	0.96	K^+	–	12
BN composite membrane	0.5: 0.01	0.772	Na^+	–	13
MoS_2 nanopore	0.1: 0.01	0.93	K^+	–	14
Holey-graphene-like membranes	1: 0.1	–	K^+	9	15
**This work**	0.1: 0.01	0.967	K^+	29	–
**This work**	0.1: 0.01	0.988	Na^+	84	–

Abbreviations: GO, graphene oxide; SNF, silk nanofiber; BP, black phosphorus; ANF, aramid nanofiber; BN, boron nitride; MoS_2, molybdenum disulfide.

Supplementary Table 2. Comparison of the power densities of nanoporous 2D membranes in the literature.

Membrane type	Thickness	Working area	Concentration gradient (M)	Power density (W m^{-2})	Ref
Oppositely charged GO	10 μm	0.8 mm^2	0.5: 0.01 NaCl	0.77	[7]
Vertical GO	354 μm	1300~8800 μm^2	0.5: 0.01 NaCl	10.6	[8]
GO/SNF composite	5 μm	0.03 mm^2	0.5: 0.01 NaCl	5.07	[9]
GO/PPSU-Py	31 μm	-	0.5: 0.01 NaCl	0.76	[16]
BP/GO multilayer	8 μm	0.03 mm^2	0.5: 0.01 NaCl	3.4	[10]
BP	8 μm	0.03 mm^2	0.5: 0.01 NaCl	1.6	[10]
MoS_2/CNF composite	4 μm	0.03 mm^2	0.5: 0.01 NaCl	5.2	[17]
Oppositely charged MXene	2 mm	0.2 mm^2	0.5: 0.01 NaCl	4.6	[12]
MXene/ANF composite	4.5 μm	0.03 mm^2	0.5: 0.01 NaCl	3.7	[11]
BN/ANF multilayer	1 μm	0.03 mm^2	0.5: 0.01 NaCl	5.9	[13]
Single-layer porous graphene	0.6 nm	1.5 um^2	1: 0.001 NaCl	27	[18]
Holey-graphene-like membranes	10 nm	3.14 um^2	1: 0.01 KCl	35	[15]
**This work**	70 nm	11.8 um^2	0.5: 0.01 NaCl	65.2	–
**This work**	70 nm	11.8 um^2	0.5: 0.01 KCl	86.2	–
**This work**	70 nm	11.8 um^2	1: 0.01 KCl	119	–

Abbreviations: GO, graphene oxide; SNF, silk nanofiber; PPSU-Py, polyphenylsulfone copolymers bearing pyridine pendants; BP, black phosphorus; MoS_2, molybdenum disulfide; CNF, cellulose nanofiber; ANF, aramid nanofiber; BN, boron nitride.

Supplementary Table 3. Simulation parameters.

Parameter	Description	Value
a	Width of the reservoir	16000 nm
b	Length of the reservoir	2000 nm
$W_{\mathrm{Si-l}}$	Width of the large opening side of silicon channel	12000 nm
$W_{\mathrm{Si-s}}$	Width of the small opening side of silicon channel	3000 nm
l_{P}	Length of 2DPI channel	70 nm
l_{Si}	Length of silicon channel	930 nm
d_{P}	Size of 2DPI channel	2.32 nm
d_{I}	Inter-channel distance	2.56 nm
D_{c}	Diffusion coefficient of K^+ ion	1.96×10^{-9} m^2 s^{-1}
D_{a}	Diffusion coefficient of Cl^- ion	2.03×10^{-9} m^2 s^{-1}
σ	Surface charge density of 2DPI channel	0.012 C m^{-2}

Supplementary References

1. Liu, H. Y. *et al.* Covalent organic frameworks linked by amine bonding for concerted electrochemical reduction of CO_2. *Chem* **4**, 1696–1709 (2018).
2. Schniepp, H. C. *et al.* Functionalized single graphene sheets derived from splitting graphite oxide. *J. Phys. Chem. B* **110**, 8535–8539 (2006).
3. Lu, M., Chen, B., He, T., Li, Y. & Tour, J. M. Synthesis, grafting, and film formation of porphyrins on silicon surfaces using triazenes. *Chem. Mater.* **19**, 4447–4453 (2007).
4. Hontorialucas, C., Lopezpeinado, A. J., Lopezgonzalez, J. D. D., Rojascervantes, M. L. & Martinaranda, R. M. Study of oxygen-containing groups in a series of graphite oxides - Physical and chemical characterization. *Carbon* **33**, 1585–1592 (1995).
5. Liu, X. *et al.* Power generation by reverse electrodialysis in a single-layer nanoporous membrane made from core-rim polycyclic aromatic hydrocarbons. *Nat. Nanotechnol.* **15**, 307–312 (2020).
6. Esfandiar, A. *et al.* Size effect in ion transport through angstrom-scale slits. *Science* **358**, 511–513 (2017).
7. Ji, J. *et al.* Osmotic power generation with positively and negatively charged 2D nanofluidic membrane pairs. *Adv. Funct. Mater.* **27**, 1603623 (2017).
8. Zhang, Z. K. *et al.* Vertically transported graphene oxide for high-performance osmotic energy conversion. *Adv. Sci.* **7**, 2000286 (2020).
9. Xin, W. *et al.* Biomimetic nacre-like silk-crosslinked membranes for osmotic energy harvesting. *ACS Nano* **14**, 9701–9710 (2020).
10. Zhang, Z. *et al.* Oxidation promoted osmotic energy conversion in black phosphorus membranes. *Proc. Natl. Acad. Sci. U.S.A.* **117**, 13959–13966 (2020).
11. Zhang, Z. *et al.* Mechanically strong MXene/Kevlar nanofiber composite membranes as high-performance nanofluidic osmotic power generators. *Nat. Commun.* **10**, 2920 (2019).
12. Ding, L. *et al.* Oppositely charged $Ti_3C_2T_x$ MXene membranes with 2D nanofluidic channels for osmotic energy harvesting. *Angew. Chem. Int. Ed.* **132**, 8798–8804 (2020).
13. Chen, C. *et al.* Bio-inspired nanocomposite membranes for osmotic energy harvesting. *Joule* **4**, 247–261 (2020).
14. Feng, J. *et al.* Single-layer MoS_2 nanopores as nanopower generators. *Nature* **536**, 197–200 (2016).
15. Wang, H. *et al.* Blue energy conversion from holey-graphene-like membranes with a high density of subnanometer pores. *Nano Lett.* **20**, 8634–8639 (2020).
16. Zhu, X. *et al.* A charge-density-tunable three/two-dimensional polymer/graphene oxide heterogeneous nanoporous membrane for ion transport. *ACS Nano* **11**, 10816–10824 (2017).
17. Zhu, C. C. *et al.* Metallic two-dimensional MoS_2 composites as high-performance osmotic energy conversion membranes. *J. Am. Chem. Soc.* **143**, 1932–1940 (2021).
18. Fu, Y. J., Guo, X., Wang, Y. H., Wang, X. W. & Xue, J. M. An atomically-thin graphene reverse electrodialysis system for efficient energy harvesting from salinity gradient. *Nano Energy* **57**, 783–790 (2019).
